# Puerarin Targets MIC19 to Suppress Mitochondrial Metabolism of Tumor‐Infiltrating Tregs and Enhance Anti‐tumor Immunity

**DOI:** 10.1002/advs.202512793

**Published:** 2025-11-18

**Authors:** Yu Li, Ziyan Song, Jianjun Ding, Yiheng Zhou, Tianning Huang, Qufei Qian, Miao Yu, Wenzhao Chen, Jiazheng Liu, Ling Lu, Qiuyang Chen

**Affiliations:** ^1^ Collaborative Innovation Center for Cancer Personalized Medicine and Hepatobiliary Center The First Hospital Affiliated with Nanjing Medical University 300 Guangzhou Road Nanjing Jiangsu 210029 China; ^2^ Department of General Surgery The Affiliated BenQ Hospital of Nanjing Medical University No. 71 Hexi Street Nanjing Jiangsu 210019 China; ^3^ Department of Plastic Surgery The Affiliated Friendship Plastic Surgery Hospital of Nanjing Medical University No. 146 Hanzhong Road Nanjing Jiangsu 210029 China; ^4^ School of Food Science and Technology Jiangnan University No. 1800, Lihu Avenue Wuxi Jiangsu 214122 China; ^5^ State Key Laboratory of Quality Research in Chinese Medicine Macau Institute for Applied Research in Medicine and Health Guangdong‐Hong Kong‐Macao Joint Laboratory of Respiratory Infectious Disease Macau University of Science and Technology Avenida Wai Long Taipa Macau 999078 China

**Keywords:** hepatocellular carcinoma, immunotherapy, mitochondrial metabolism, puerarin, Regulator T cells

## Abstract

Regulatory T cells (Tregs) are pivotal mediators of immunosuppression in hepatocellular carcinoma, but strategies for selectively disrupting their function remain underdeveloped. Here, puerarin, a natural isoflavone is identifed as a selective immunometabolic modulator. It impairs mitochondrial metabolism in tumor‐infiltrating Tregs (Ti‐Tregs) without affecting conventional T cells. Mechanistically, puerarin directly binds to MIC19—a core subunit of the mitochondrial contact site and cristae organizing system—leading to its degradation and disruption of the MIC19–MIC60 complex. This disruption causes cristae disorganization, reduces oxidative phosphorylation, and weakens the immunosuppressive function of Ti‐Tregs. In vivo, puerarin decreases Ti‐Treg infiltration, thereby enhancing antitumor immunity without causing systemic toxicity. Furthermore, MIC19 knockdown and site‐directed mutagenesis studies validate the role of critical MIC19 residues (His180, Gln187, and Tyr211) in puerarin's activity. These results reveal a mechanism by which puerarin suppresses mitochondrial metabolism of Ti‐Tregs and emphasize the therapeutic potential of natural compounds in metabolic targeting for cancer immunotherapy.

## Graphical abstract

Puerarin targets mitochondrial MIC19 in tumor‐infiltrating Tregs, impairing their immunosuppressive function and enhancing antitumor immunity. This study uncovers the metabolic dependency of tumor‐infiltrating Tregs and highlights a potential metabolic‐targeted immunotherapeutic strategy for liver cancer.

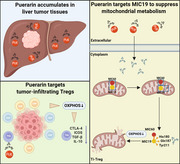

## 1 Introduction

Hepatocellular carcinoma (HCC), a malignancy with persistently low 5‐year survival rates (<20%), remains a formidable global health challenge.^[^ ^1^, ^2^ ^]^ Although immune checkpoint inhibitors targeting the programmed death‐1/programmed death ligand 1 axis have broadened therapeutic options, acquired resistance and limited durability of treatment responses underscore the pressing need to overcome the immunosuppressive tumor microenvironment (TME).^[^ ^3^, ^4^ ^]^ Notably, regulatory T cells (Tregs) are pathologically enriched in HCC TME, orchestrating immune evasion through direct suppression of effector T cells and leading to poor prognosis.^[^ ^5^, ^6^ ^]^ Current Treg‐targeting strategies, such as anti‐CD25 antibodies and PI3K inhibitors, are clinically limited by systemic immunosuppression and off‐tumor toxicity.^[^ ^7^, ^8^ ^]^ These therapeutic challenges highlight the need for TME‐specific interventions that selectively modulate Treg function.

Emerging evidence reveals that tumor‐infiltrating Tregs (Ti‐Tregs) exhibit unique metabolic dependencies compared with their peripheral counterparts, particularly their reliance on mitochondrial oxidative phosphorylation (OXPHOS) to sustain immunosuppressive functions and FOXP3 stability within the nutrient‐deprived TME.^[^ ^12^, ^13^ ^]^ In contrast, effector T cells depend primarily on glycolysis,^[^ ^14^, ^15^ ^]^ revealing a metabolic divergence that presents an opportunity for selective Treg modulation. However, clinically translatable small‐molecule agents capable of precise metabolic targeting remain elusive, especially those derived from natural products with proven safety profiles.

Puerarin, a bioactive isoflavone extracted from Pueraria lobata, has demonstrated pleiotropic therapeutic activities—including anti‐inflammatory, antioxidant, and cardiovascular protective effects.^[^ ^16^, ^17^, ^18^ ^]^ It has been widely used in traditional medicine and demonstrates excellent clinical safety profiles.^[^ ^19^ ^]^ More recently, puerarin has also shown promise in tumor‐related studies, where it was reported to inhibit proliferation, induce apoptosis, and modulate inflammatory cytokine expression.^[^ ^20^, ^21^ ^]^ However, the immunological impact of puerarin, particularly on tumor‐infiltrating immune cells such as Tregs, and the underlying immunomodulatory mechanisms have not been comprehensively explored.

In this study, we hypothesized that puerarin selectively impairs the mitochondrial metabolic adaptation of Ti‐Tregs by targeting MIC19, thereby destabilizing their suppressive function and ultimately enhancing anti‐tumor immunity.

## 2 Results

### 2.1 Puerarin Inhibits HCC Progression via Targeting Tumor‐Infiltrating Tregs

**Figure 1:**
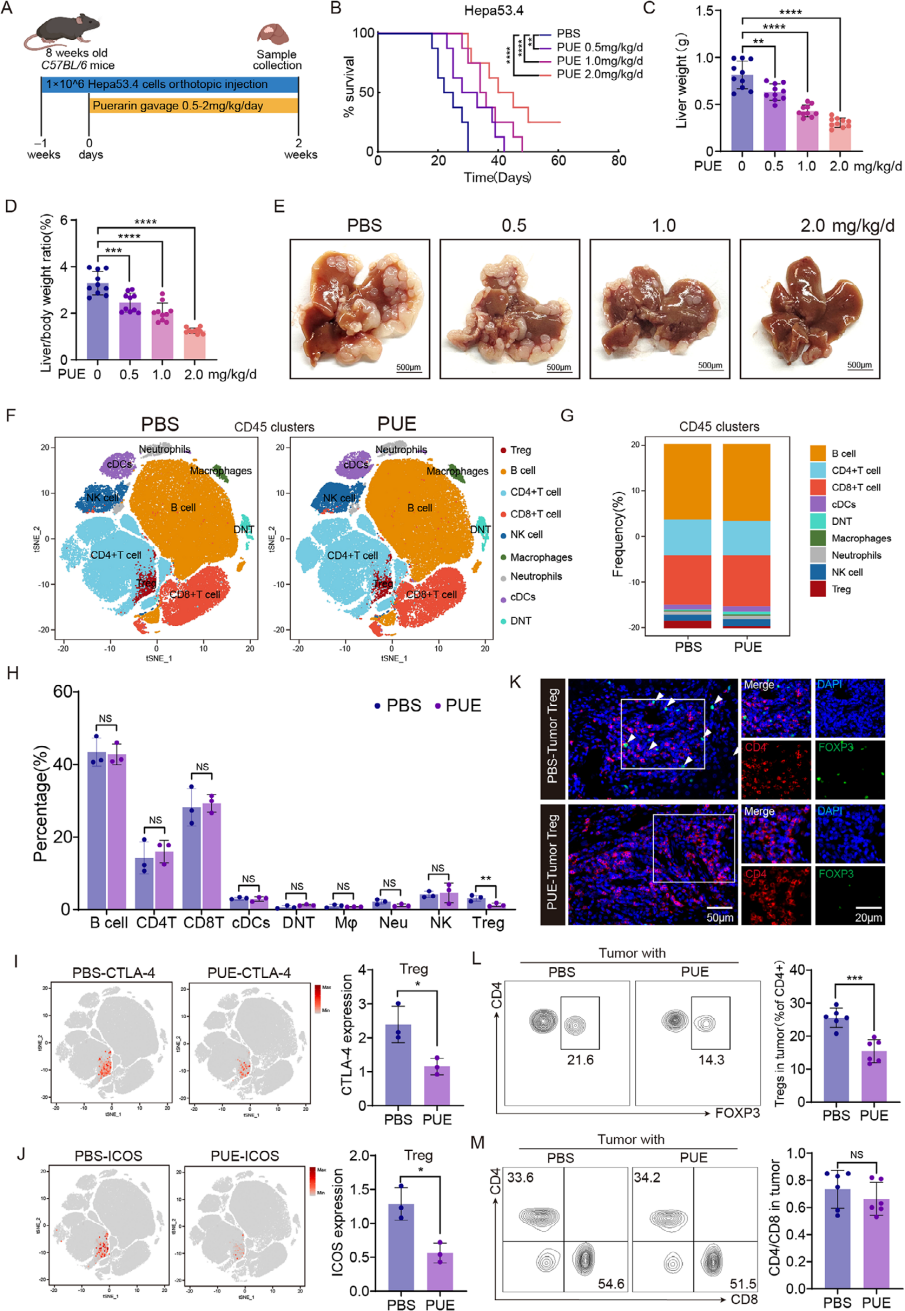
Puerarin treatment suppresses hepatocellular carcinoma (HCC) progression and regulates immune cells in C57BL/6 mice. A) Experimental scheme: C57BL/6 mice were orthotopically injected with 1 × 10⁶ Hepa53.4 cells and treated daily with an oral gavage of puerarin (PUE) (0.5–2.0 mg kg^−1^day^−1^), starting one‐week post‐injection. B) Kaplan–Meier survival curves of HCC model mice treated with PBS or PUE (n = 10) (PBS vs PUE 0.5 mg kg^−1^day^−1^: hazard ratio [HR] = 0.26, 95% confidence interval [CI] = 0.17–0.40; PBS vs PUE 1.0 mg kg^−1^day^−1^: HR = 0.14, 95% CI = 0.048–0.43; PBS vs PUE 2.0 mg kg^−1^day^−1^: HR = 0.059, 95% CI = 0.021–0.17). C) Liver weight of the HCC model mice at experimental endpoint in each treatment group (n = 10). D) Liver‐to‐body weight ratios of the HCC model mice in each treatment group (n = 10). E) Representative images of HCC from 3 weeks after treatment with different doses of PUE. F) T‐distributed stochastic neighbor embedding (T‐SNE) plots of CD45⁺ immune cells from the HCC model mice analyzed by mass cytometry (CyTOF). Nine immune clusters were identified, including regulatory T cells (Tregs), B cells, CD4⁺ T cells, CD8⁺ T cells, natural killer (NK) cells, macrophages, neutrophils, conventional dendritic cells (cDCs), and double‐negative T cells (DNTs). G,H) Frequency (G) and Quantification (H) of each CD45⁺ immune cell subset in HCC model mice treated with PBS or PUE (2.0 mg kg^−1^day^−1^) (n = 3). I,J) CTLA‐4 (I) and ICOS (J) expression in tumor‐infiltrating Tregs (Ti‐Tregs) obtained from the HCC model mice analyzed via CyTOF. Representative t‐SNE feature plots are demonstrated along with quantification of median fluorescence intensity (n = 3). K) Immunofluorescence staining of CD4⁺FOXP3⁺ Treg infiltration in HCC model mice treated with PBS or PUE (2.0 mg kg^−1^day^−1^) (n = 3). Scale bars: overview, 50 µm; inset = 20 µm. L,M) Flow cytometry analysis of Ti‐Tregs (L) and CD4⁺/CD8⁺ T cell ratios (M) in HCC model mice treated with PBS or PUE (2.0 mg kg^−1^day^−1^) (n = 6). Data are presented as mean ± standard error of the mean (SEM)*. P*‐values were calculated using a log‐rank test for survival analysis in (B) and an unpaired two‐tailed Student's t‐test in (C–D), (H–J), and (L–M). ^*^ *P* < 0.05; ^**^ *P* < 0.01; ^***^ *P* < 0.001; ^****^ *P* < 0.0001.

We first assessed the therapeutic efficacy of puerarin in a murine HCC model by orthotopically implanting Hepa53.4 cells into C57BL/6 mice, followed by daily oral gavage of puerarin at doses ranging from 0.5 to 2 mg kg^−1^ starting one week after tumor inoculation (Figure **1**A). Kaplan–Meier survival analysis revealed that puerarin significantly prolonged mouse survival in a dose‐dependent manner (Figure 1B). Tumor burden, assessed by liver tumor volume and weight, was also markedly reduced following puerarin treatment, with a clear dose‐dependent relationship (Figure 1C–E).

To investigate how puerarin modulates the tumor immune microenvironment, we treated mice with 2 mg kg^−1^ day^−1^ puerarin and analyzed CD45⁺ tumor‐infiltrating immune cells using mass cytometry (CyTOF). Dimensionality reduction via t‐distributed stochastic neighbor embedding identified nine major immune cell subsets, including Tregs, B cells, CD4⁺ T cells, CD8⁺ T cells, natural killer cells, macrophages, neutrophils, conventional dendritic cells, and double‐negative T cells (Figure 1F). Puerarin treatment significantly decreased the proportion of Ti‐Tregs (Figure 1G,H) and downregulated the expression of the immunosuppressive markers CTLA‐4 and ICOS (Figure 1I,J), indicating an inhibitory effect on both Treg infiltration and function.

To investigate the in vivo distribution and biosafety profile of puerarin, we conjugated puerarin to a CY7 fluorescent probe (PUE‐CY7) and performed whole‐body imaging after oral gavage. PUE‐CY7 accumulated preferentially in the liver within 1–3 h post‐administration (Figure S1A, Supporting Information), and ex vivo imaging confirmed a higher fluorescence signal in the liver tumor and colon tissues compared with other organs (Figure S1B, Supporting Information). Quantitative analysis demonstrated significantly elevated puerarin concentrations in the liver and colon, but not in the blood, lung, or kidney (Figure S1C, Supporting Information). Importantly, TUNEL staining showed increased apoptotic cells in the liver tissues after puerarin treatment, but without evident cytotoxicity in normal liver, lung, colon, or kidney tissues (Figure S1D,E, Supporting Information). Serum alanine transaminase and aspartate transaminase levels were reduced upon puerarin treatment (Figure S1F, Supporting Information), and no significant changes were observed in blood urea nitrogen or serum creatinine levels (Figure S1G, Supporting Information), indicating a favorable safety profile. These data support the preferential accumulation of puerarin in liver tumors, where it exerts anti‐tumor effects without causing off‐target toxicity.

Next, we assessed whether puerarin directly targets tumor cells. In vitro colony formation, wound healing, and transwell invasion assays revealed no significant changes in the proliferation or invasiveness of Hepa53.4 cells across a range of puerarin concentrations (Figure S2A–F, Supporting Information). Furthermore, puerarin treatment did not improve survival or reduce tumor growth in *Rag1^−^/^−^* immunodeficient mice bearing Hepa53.4 tumors (Figure S2G–K, Supporting Information), indicating that its antitumor effects are not mediated by direct cytotoxicity, but rather by modulation of the immune microenvironment.

To further validate the role of Ti‐Tregs in mediating puerarin's antitumor activity, immunofluorescence and flow cytometry showed a significant reduction in Ti‐Tregs (Figure 1K,L), whereas the proportions of CD4⁺ and CD8⁺ T cells remained largely unchanged (Figure 1M). Notably, in *FOXP3‐DTR* mice, where Tregs can be selectively depleted using diphtheria toxin (Figure S3A, Supporting Information), the antitumor effects of puerarin were abolished. Treg‐depleted mice showed no significant improvement in tumor progression or immune reprogramming upon puerarin treatment (Figure S3B–G, Supporting Information). Collectively, these findings demonstrate that puerarin suppresses HCC progression primarily by inhibiting the infiltration and immunosuppressive activity of Ti‐Tregs.

### 2.2 Puerarin Inhibits Ti‐Treg Differentiation, Proliferation, and Suppressive Function

To further investigate the regulatory effects of puerarin on Ti‐Tregs, we analyzed immune cell compositions in the spleen, peripheral blood, and lymph nodes of the murine HCC model mice treated with 2 mg kg^−1^ day^−1^ puerarin (Figure S4A, Supporting Information). Flow cytometry revealed that the frequencies of Tregs remained unchanged across the immune compartments, with no significant alterations in CD4⁺ and CD8⁺ T cell populations (Figure S4B, C, Supporting Information).

**Figure 2:**
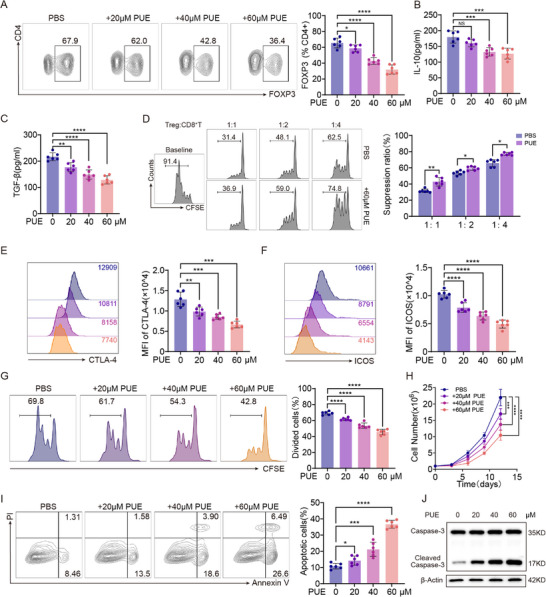
Puerarin (PUE) inhibits tumor‐infiltrating regulatory T cells (Ti‐Treg) function and differentiation in vitro. A) FOXP3 expression in naïve CD4⁺ T cells isolated from mice spleens and co‐cultured with Hepa53.4 cells following stimulation with anti‐CD3/CD28 magnetic beads and interleukin (IL‐2), in the presence of increasing concentrations of PUE (0, 20, 40, 60 µm) for 3 days (n = 6). (B–C) IL‐10 (B) and transforming growth factor (TGF)‐β (C) levels in mice Ti‐Tregs from (A) culture supernatants were measured by enzyme‐linked immunosorbent assay (ELISA) following increasing concentrations of PUE treatment (0, 20, 40, 60 µm) for 3 days (n = 6). D) Suppression assay: CD8⁺ T cells from C57BL/6 mice splenic T cells were labeled with CFSE and co‐cultured with mice Ti‐Tregs pre‐treated with PBS or PUE (60 µm) at the indicated Treg:CD8 ratios for 3 days. Proliferation was measured by flow cytometry (n = 6). E,F) CTLA‐4 (E) and ICOS (F) expression on mice Ti‐Tregs from (A) treated with increasing concentrations of PUE (0, 20, 40, 60 µm). Flow cytometry results are shown as a histogram and mean fluorescence intensity (MFI) quantification (n = 6). G) Proliferation of CFSE‐labeled mice Ti‐Tregs from (A) cultured with increasing PUE concentrations for 3 days. H) Ti‐Tregs from (A) growth curves over 14 days with indicated PUE concentrations (0, 20, 40, 60 µm) (n = 6). I) Flow cytometric staining of apoptotic mice Ti‐Tregs treated with PUE (0, 5, 10, and 20 mM) for 3 days (n = 6). J) Caspase‐3 and cleaved Caspase‐3 expression in mice Ti‐Tregs treated with PUE (0, 5, 10, and 20 mM) for 3 days (n = 6). Data are presented as mean ± standard error of the mean (SEM). *P*‐values were calculated using unpaired two‐tailed Student's *t*‐test in (A–G) and one‐way analysis of variance (ANOVA) with Tukey's multiple comparisons in (H) and (I). ^*^ *P* < 0.05; ^**^ *P* < 0.01; ^***^ *P* < 0.001; ^****^ *P* < 0.0001.

To elucidate the direct action of puerarin on Tregs in vitro, we first established its half‐maximal inhibitory concentration: 60 µM yielded the strongest effect on Treg activity (Figure S4D, Supporting Information). CD4⁺ naïve T cells were isolated from C57BL/6 mouse spleens, activated with anti‐CD3/CD28 magnetic beads in the presence of interleukin (IL)‐2, and then co‐cultured with the murine HCC cell line Hepa53.4 to recapitulate the TME (Figure S4E, Supporting Information). Under these conditions, puerarin curtailed Ti‐Treg differentiation in a dose‐dependent manner (Figure **2**A) and significantly reduced their secretion of the immunosuppressive IL‐10 and transforming growth factor (TGF)‐β (Figure 2B, C).

To assess the functional consequences on Ti‐Tregs, we co‐cultured them with CFSE‐labeled CD8⁺ T cells at varying ratios. Puerarin‐treated Ti‐Tregs (60 µm) exhibited markedly impaired suppressive activity against CD8⁺ T cell proliferation (Figure 2D), along with reduced expression of the immunosuppressive markers CTLA‐4 and ICOS (Figure 2E, F).

In addition, we evaluated the impact of puerarin on Ti‐Treg proliferation and apoptosis. CFSE‐based proliferation assays and direct cell counting demonstrated that puerarin significantly inhibited Ti‐Treg expansion (Figure 2G, H). Concurrently, Annexin V/PI staining showed that puerarin treatment led to a marked increase in Ti‐Treg apoptosis (Figure 2I), which was further corroborated by enhanced expression of cleaved Caspase‐3, as detected by Western blot analysis (Figure 2J). Taken together, these results indicate that puerarin not only impairs Ti‐Treg induction and proliferation but also markedly weakens its immunosuppressive function in the TME.

### 2.3 Puerarin Impairs Mitochondrial Function and Oxidative Phosphorylation in Ti‐Tregs

**Figure 3:**
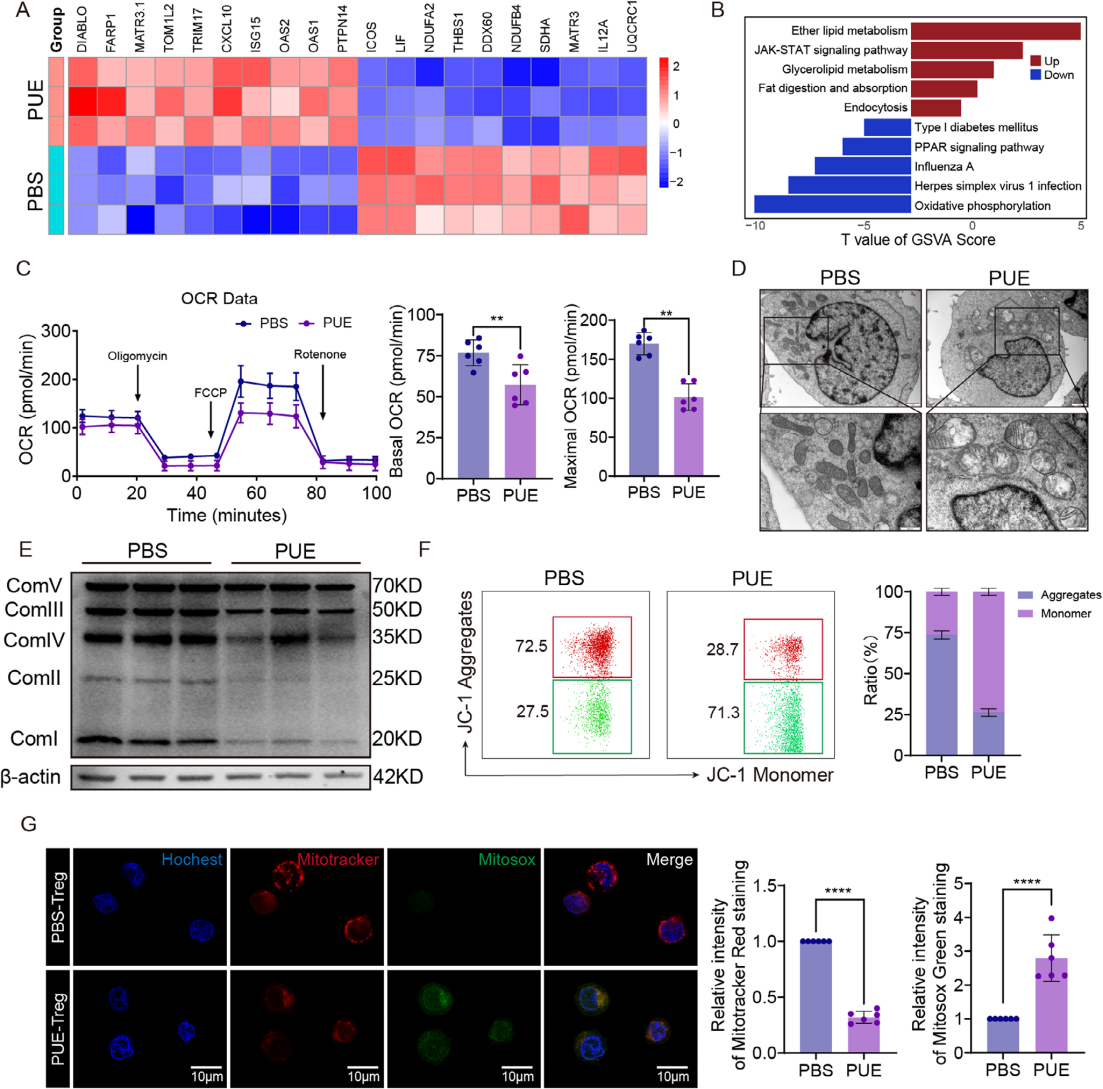
Puerarin (PUE) alters gene expression and mitochondrial function. A) Heatmap showing the top 10 differentially expressed genes (DEGs) in mice tumor‐infiltrating regulatory T cells (Ti‐Tregs) treated with PBS or PUE (2.0 mg kg^−1^day^−1^), based on bulk RNA sequencing (n = 3). B) Gene set variation analysis (GSVA) of pathways altered in mice Ti‐Tregs treated with PBS or PUE (2.0 mg kg^−1^day^−1^) (n = 3). C) Oxygen consumption rate (OCR) measured by Seahorse XF Analyzer in mice Ti‐Tregs. Basal and maximal OCR were quantified and compared (n = 6). D) Transmission electron microscopy (TEM) images of mice Ti‐Tregs treated with PBS or PUE (2.0 mg kg^−1^day^−1^), showing mitochondrial morphology at both low and high magnification (scale bars: 1 µm and 500 nm). E) Western blot analysis of mitochondrial respiratory chain complex subunits (Complex I–V) in mice Ti‐Tregs treated with PBS or PUE (2.0 mg kg^−1^day^−1^) (n = 3). F) Flow cytometry analysis of mitochondrial membrane potential using JC‐1 staining in mice Ti‐Tregs treated with PBS or PUE (2.0 mg kg^−1^day^−1^). The ratio of JC‐1 aggregates (red) to monomers (green) was calculated (n = 6). G) Confocal imaging of mice Ti‐Tregs stained with Hoechst (nucleus), Mitotracker Red (mitochondria), and MitoSOX Green (mitochondrial reactive oxygen species [ROS]) following treatment with PBS or PUE (2.0 mg kg^−1^day^−1^). Representative images and quantitative fluorescence intensity analysis are shown (n = 6) (Scale bars = 10 µm). Data are presented as mean ± standard error of the mean (SEM). P‐values were calculated using an unpaired two‐tailed Student's *t*‐test in (C) and (G). ^*^ *P* < 0.05; ^**^ *P* < 0.01; ^***^ *P* < 0.001; ^****^ *P* < 0.0001.

To further explore the potential mechanisms underlying puerarin‐mediated modulation of Ti‐Treg function, we isolated Ti‐Tregs from the murine HCC model mice treated with 2 mg kg^−1^ day^−1^ puerarin using a flow cytometry‐based sorting strategy (Figure S4F, Supporting Information) and performed transcriptomic analysis of Ti‐Tregs. Volcano plot analysis revealed numerous differentially expressed genes between the puerarin‐treated and control groups (Figure S5A, Supporting Information). Heatmap visualization demonstrated that many downregulated genes were enriched in components of the mitochondrial electron transport chain (Figure **3**A). Gene set variation analysis further indicated that oxidative phosphorylation was the most significantly downregulated pathway (Figure 3B), suggesting that puerarin may suppress the immunosuppressive function of Ti‐Tregs by inhibiting their mitochondrial activity.

To validate this hypothesis, we assessed the metabolic profile of Ti‐Tregs following puerarin treatment. Seahorse analysis revealed a marked reduction in oxygen consumption rate (OCR) (Figure 3C). However, extracellular acidification rate (ECAR), indicative of glycolytic activity, was moderately increased (Figure S5B, C, Supporting Information), suggesting a potential compensatory shift toward glycolysis in response to impaired oxidative phosphorylation. Transmission electron microscopy (TEM) further showed substantial mitochondrial alterations in puerarin‐treated Ti‐Tregs, including reduced mitochondrial numbers, swelling, and disrupted cristae structure (Figure 3D; Figure S5D–F, Supporting Information). These morphological changes were accompanied by a significant decrease in intracellular adenosine triphosphate (ATP) levels (Figure S5G, Supporting Information), supporting a functional impairment of mitochondrial energy production.

Next, we examined the expression of mitochondrial complex–related proteins and found that puerarin treatment led to a broad downregulation of components across multiple respiratory complexes (Figure 3E). Moreover, JC‐1 staining revealed a decrease in mitochondrial membrane potential, whereas MitoSOX staining showed elevated mitochondrial levels of reactive oxygen species in puerarin‐treated Tregs, indicative of pronounced mitochondrial stress and damage (Figure 3G; Figure S5H, Supporting Information). To determine whether puerarin exerts a selective effect on Ti‐Tregs, we further examined its impact on tumor‐infiltrating CD8⁺ T cells (Ti‐CD8⁺). We co‐cultured murine CD8⁺ T cells with Hepa53.4 cells to mimic the TME (Figure S5I, Supporting Information). Notably, puerarin treatment did not significantly induce apoptosis in Ti‐CD8⁺ cells nor did it elevate the levels of cleaved caspase‐3 (Figure S5J, K, Supporting Information). However, Seahorse‐based analysis revealed that puerarin suppressed OXPHOS in Ti‐CD8⁺ cells (Figure S5L, Supporting Information). Despite this result, the effector functions of Ti‐CD8⁺ cells, including Granzyme B and interferon‐γ production, remained unaffected (Figure S5M, N, Supporting Information), likely because CD8⁺ T cells primarily rely on glycolysis rather than OXPHOS for their cytotoxic activity.

To better understand why Ti‐Tregs are particularly sensitive to OXPHOS‐targeted metabolic disruption, we analyzed public single‐cell transcriptomic data from liver cancer tissues (GSE162616), focusing on paired tumor and adjacent normal samples. Dimensionality reduction and clustering identified distinct tumor‐infiltrating T‐cell subsets, including CD8⁺ Tex cells, CD4⁺ T cells, CD8⁺ T cells, Tregs, and γδ T cells (Figure S6A–D, Supporting Information). Among these immune subsets, metabolic pathway scoring revealed that Ti‐Tregs exhibited the highest dependency on OXPHOS (Figure S6E, F, Supporting Information), which was further supported by Kyoto Encyclopedia of Genes and Genomes and Gene Set Enrichment Analysis (Figure S6G, H, Supporting Information). Taken together, these findings suggest that puerarin impairs Ti‐Treg immunosuppressive activity by inducing mitochondrial dysfunction and suppressing oxidative phosphorylation, thereby exerting its immunoregulatory effects.

### 2.4 Identification of Direct Protein Targets of Puerarin in Ti‐Tregs

**Figure 4:**
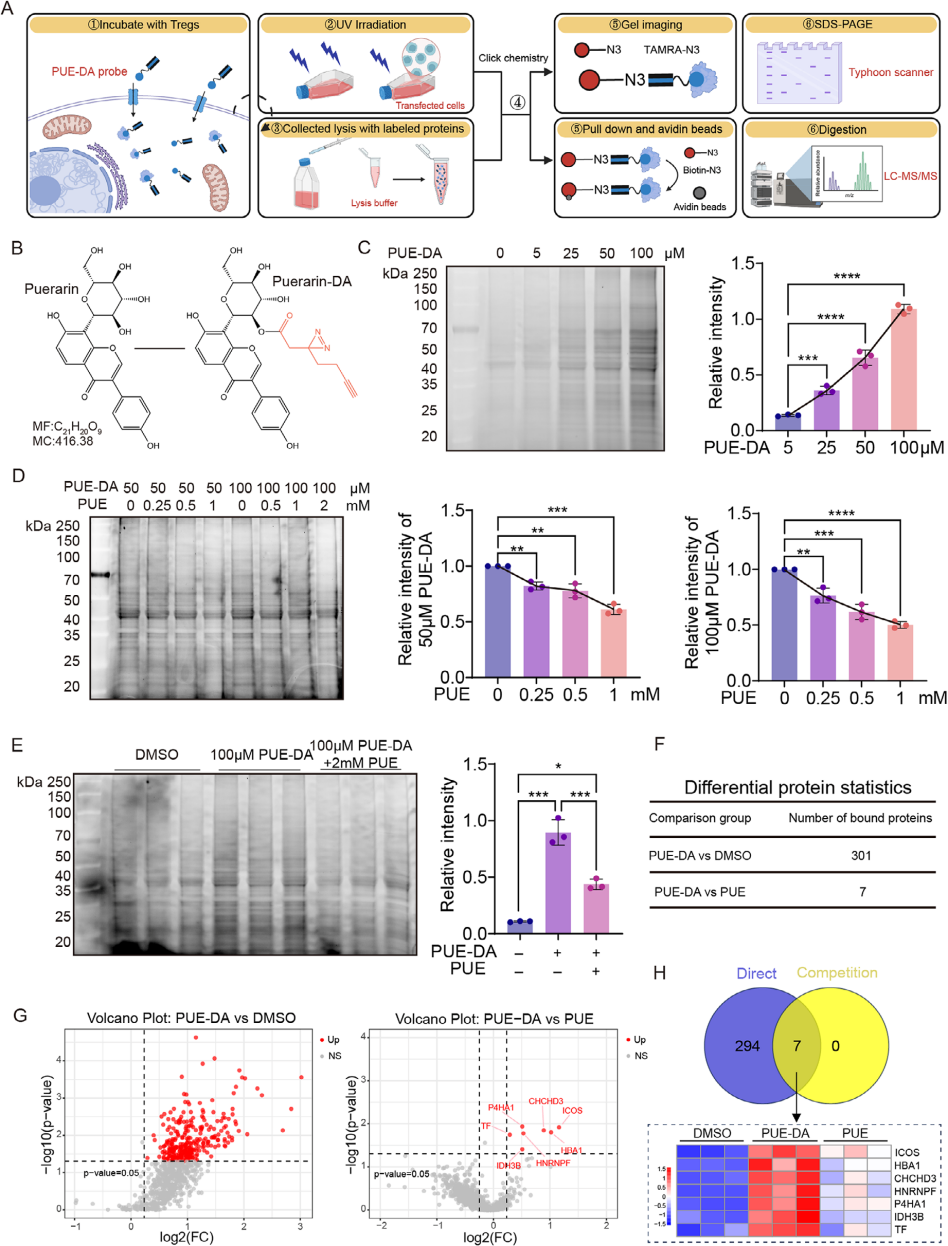
Identification of direct protein targets of puerarin (PUE) in tumor‐infiltrating regulatory T cells (Ti‐Tregs). A) Schematic workflow of the photoaffinity labeling and protein target identification using the PUE‐originated probe (PUE‐DA) in mice Ti‐Tregs, followed by ultraviolet (UV) crosslinking, click chemistry, sodium dodecyl‐sulfate polyacrylamide gel electrophoresis (SDS‐PAGE) visualization, pull‐down, and liquid chromatography‐tandem mass spectrometry (LC‐MS/MS). B) Chemical structures of PUE and the photoaffinity‐based probe PUE‐DA containing a diazirine group for UV activation and a terminal alkyne for click chemistry labeling. C) Fluorescence imaging of labeled proteins after treatment of mice Ti‐Tregs with increasing concentrations of PUE‐DA (0–100 µm) for 24 h, followed by click labeling and SDS‐PAGE. D) Competitive binding assay of labeled proteins after treating mice Ti‐Tregs with PUE‐DA (50 or 100 µm) and escalating doses of PUE (0–2 mM) for 24 h to evaluate probe competition (n = 3). E) Fluorescence imaging of labeled proteins of mice Ti‐Tregs treated with dimethyl sulfoxide (DMSO), PUE‐DA (100 µm), or PUE‐DA plus PUE (2 mM). Quantification shows competitive inhibition of labeling by excess free PUE (n = 3). F) Summary of MS results showing the number of proteins identified in PUE‐DA vs DMSO (301 proteins) and the subset of competitive targets significantly reduced by co‐treatment with PUE (7 proteins). G) Volcano plots of LC‐MS/MS data showing significantly enriched proteins in PUE‐DA vs DMSO (left) and competitive reduction of protein binding in PUE‐DA vs PUE co‐treatment (right). Candidate direct binding proteins include ICOS, HBA1, CHCHD3, HNRNPF, P4HA1, IDH3B, and TF (n = 3). H) Venn diagram showing the overlap between direct‐binding proteins (PUE‐DA vs DMSO) and competition‐validated targets (PUE‐DA vs PUE). The heatmap displays the abundance of the 7 candidate targets across the conditions. Data are presented as mean ± standard error of the mean (SEM). P‐values were calculated using one‐way analysis of variance (ANOVA) with Tukey's multiple comparisons in (C–E). ^*^ *P* < 0.05; ^**^ *P* < 0.01; ^***^ *P* < 0.001; ^****^ *P* < 0.0001.

To further elucidate the molecular mechanism by which puerarin regulates Ti‐Treg function, we employed an affinity‐based chemoproteomic approach to identify its direct protein targets in Ti‐Tregs (Figure **4**A). We designed and synthesized a photo‐reactive chemical probe based on puerarin, incorporating a diazirine group for ultraviolet‐induced covalent crosslinking at 365 nm and a terminal alkyne moiety for subsequent click chemistry–based labeling (Figure 4B).

We first evaluated the probe's capacity to capture intracellular binding proteins under live‐cell conditions. Ti‐Tregs were incubated with increasing concentrations of the puerarin‐derived probe (PUE‐DA) for 24 h, followed by a copper‐catalyzed azide–alkyne cycloaddition reaction with azide‐rhodamine. Fluorescent gel imaging revealed a concentration‐dependent increase in protein labeling, indicating efficient intracellular protein capture by the probe (Figure 4C). Competitive binding assays were then performed by co‐incubating cells with fixed concentrations of PUE‐DA and increasing amounts of free puerarin. A gradual decrease in fluorescence intensity was observed with high concentrations of free puerarin, suggesting that puerarin competes with the probe for protein binding and confirming its specific interaction with intracellular proteins (Figure 4D).

Building upon these results, we performed liquid chromatography‐tandem mass spectrometry–based proteomic profiling of Ti‐Tregs treated with DMSO (vehicle control), 100 µm PUE‐DA, or 100 µm PUE‐DA combined with 2 mM free puerarin for 24 h. After confirming labeling efficiency via fluorescent gel imaging (Figure 4E), samples were processed for proteomic analysis. A total of 301 candidate binding proteins were identified in the PUE‐DA‐treated group, among which seven proteins showed significant signal reduction upon free puerarin competition, suggesting specific binding (Figure 4F). Volcano plot analysis highlighted these competitively displaced proteins (Figure 4G), and Venn diagram comparison confirmed that all seven proteins belonged to the subset of direct binders, further supporting their specificity (Figure 4H). The final list of high‐confidence targets included ICOS, HBA1, MIC19, HNRNPF, P4HA1, IDH3B, and TF. Together, these findings provide a set of candidate proteins that may mediate puerarin's immunoregulatory effects on Ti‐Tregs, offering important leads for downstream mechanistic investigations.

### 2.5 Puerarin Targets MIC19 to Inhibit Mitochondrial Function in Ti‐Tregs

Among the seven candidate targets, functional enrichment analysis highlighted MIC19—a core component of the mitochondrial contact site and cristae organizing system (MICOS)—as closely associated with oxidative phosphorylation^[^ ^22^, ^23^ ^]^ (Figure S7A, Supporting Information). As a critical regulator of cristae architecture, MIC19 is essentially involved in maintaining mitochondrial integrity and energy metabolism, and its dysfunction can lead to metabolic collapse. These findings provide a mechanistic clue that puerarin may exert its immunomodulatory effects through MIC19.

**Figure 5:**
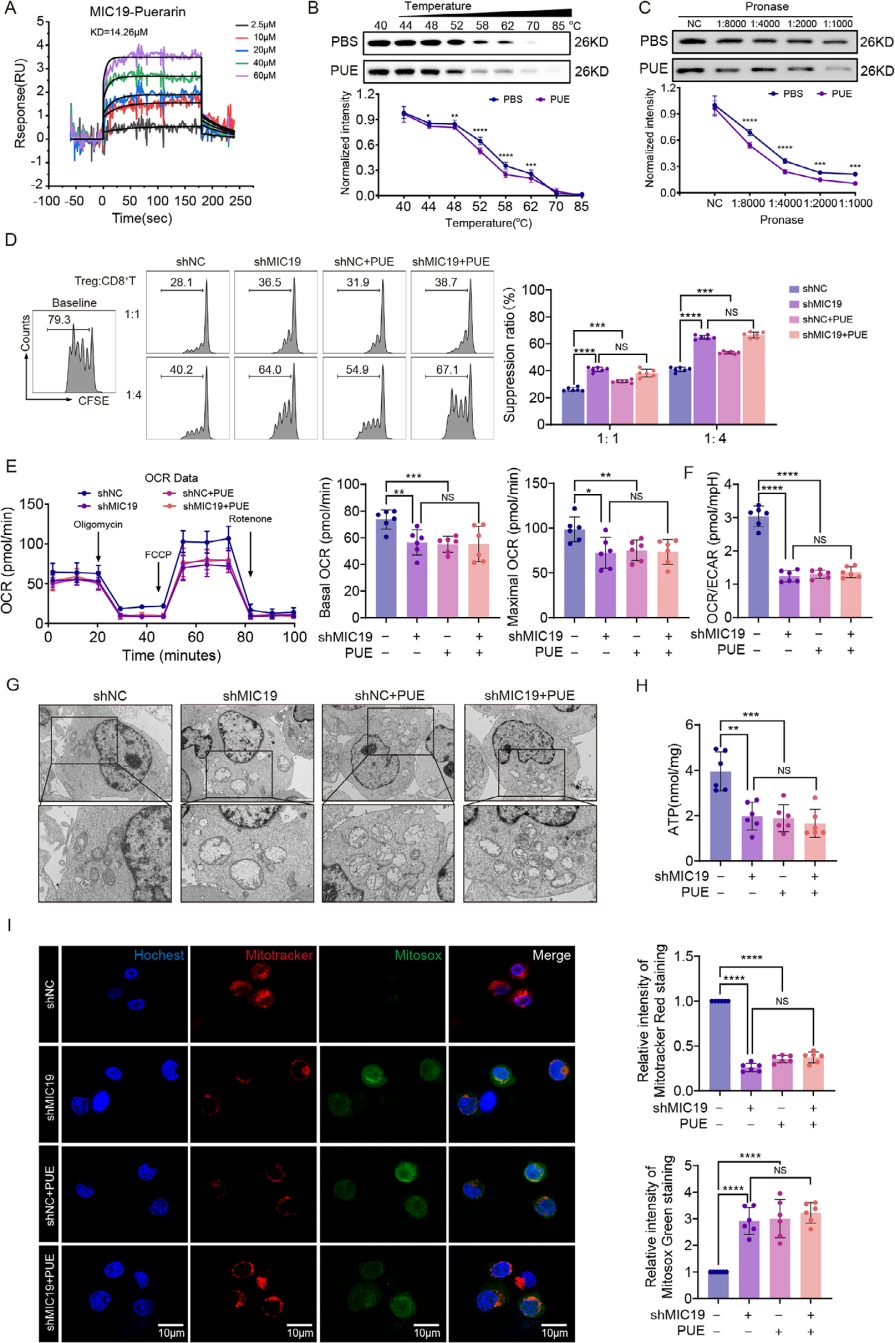
MIC19 is a direct binding target for puerarin (PUE) in regulating mitochondrial function in tumor‐infiltrating regulatory T cells (Ti‐Tregs). A) Binding affinity between MIC19 and PUE measured by surface plasmon resonance (SPR) at indicated concentrations (2.5–60 µm). B) Cellular thermal shift assay (CETSA) of MIC19 protein in mice Ti‐Tregs treated with PBS or PUE (2.0 mg kg^−1^day^−1^) across a range of temperatures. Protein band intensity was normalized to 40 °C (n = 3). C) Drug affinity responsive target stability (DARTS) assay of MIC19 protein in mice Ti‐Tregs treated with PBS or PUE (2.0 mg kg^−1^day^−1^) and increasing concentrations of pronase (n = 3). D) Suppression assay: splenic CD8⁺ T cells from C57BL/6 mice were labeled with CFSE and co‐cultured with shNC or shMIC19 natural Tregs (nTregs) pre‐treated with PBS or PUE (60 µm) at indicated Treg:CD8 ratios for 3 days. Proliferation was measured by flow cytometry (n = 6). All cells were co‐cultured with Hepa53.4 cells prior to the assay to mimic the tumor immune microenvironment. E) Oxygen consumption rate (OCR) of shNC or shMIC19 nTregs treated with PBS or PUE (60 µM) and co‐cultured with Hepa53.4 cells measured by Seahorse XF Analyzer. Basal and maximal OCR were quantified and compared (n = 6). F) OCR/extracellular acidification rate (ECAR) ratio of mice nTregs from each group (n = 6). G) Transmission electron microscopy (TEM) images of mitochondrial ultrastructure in shNC and shMIC19 nTregs treated with PBS or PUE (60 µM) and co‐cultured with Hepa53.4 cells (n = 6). H) Intracellular adenosine triphosphate (ATP) levels in shNC and shMIC19 nTregs treated with PBS or PUE (60 µm) and co‐cultured with Hepa53.4 cells measured by ATP assay (n = 6). I) Confocal imaging of shNC and shMIC19 nTregs stained with Hoechst (nucleus), Mitotracker Red (mitochondria), and MitoSOX Green (mitochondrial reactive oxygen species [ROS]) following treatment with PBS or PUE (60 µm) and co‐cultured with Hepa53.4 cells. Representative images and quantitative fluorescence intensity analysis are shown (n = 6). Scale bars = 10 µm. Data are presented as mean ± standard error of the mean (SEM). P‐values were calculated using unpaired two‐tailed Student's *t*‐test in (B–C) and one‐way analysis of variance (ANOVA) with Tukey's multiple comparisons in (D–F) and (H–I). ^*^ *P* < 0.05; ^**^ *P* < 0.01; ^***^ *P* < 0.001; ^****^ *P* < 0.0001.

To validate the direct interaction between puerarin and MIC19, we performed surface plasmon resonance analysis, which revealed high‐affinity binding with a dissociation constant (KD) of 14.26 µm (Figure **5**A). Cellular thermal shift assays (CETSA) showed that puerarin treatment reduced the thermal stability of MIC19 (Figure 5B), whereas drug affinity responsive target stability (DARTS) assays demonstrated increased protease sensitivity upon puerarin binding (Figure 5C). Together, these results confirm that puerarin directly interacts with MIC19 and may promote its destabilization and degradation.

To determine the functional importance of MIC19 in mediating puerarin's effects, we isolated natural Tregs (nTregs) from mice spleen and generated MIC19‐deficient nTregs via lentiviral short hairpin RNA (shRNA) knockdown (Figure S7B, Supporting Information). Notably, the inhibitory effects of puerarin on the production of immunosuppressive cytokines—IL‐10 and TGF‐β—were largely abrogated in MIC19‐knockdown nTregs co‐cultured with Hepa53.4 cells (Figure S7C, D, Supporting Information). Furthermore, puerarin‐mediated suppression of immune checkpoint molecules (ICOS and CTLA‐4), inhibition of Treg proliferation, and reduction in suppressive function toward CD8⁺ T cells were all significantly diminished after MIC19 knockdown (Figure S7E–G, Supporting Information; Figure 5D), supporting MIC19 as a key mediator of puerarin's immunoregulatory activity.

Next, we assessed the metabolic consequences of MIC19 depletion on puerarin‐treated Ti‐Tregs. Seahorse analysis revealed that MIC19 knockdown markedly suppressed OCR, and puerarin treatment failed to alter either OCR or ECAR in MIC19‐deficient cells (Figure 5E, F; Figure S7H, Supporting Information), suggesting a loss of metabolic responsiveness to puerarin in the absence of its target. Consistent findings from TEM, ATP quantification, JC‐1 staining, MitoSOX, and apoptosis assays further supported the role of MIC19 in mediating puerarin's effects on mitochondrial structure and function (Figure 5G–I; Figure S7I–K, Supporting Information).

To determine whether the effect of puerarin on Ti‐Tregs is conserved in human cells, we isolated high‐purity nTregs from healthy donors’ peripheral blood using magnetic bead sorting for CD4⁺CD25⁺CD127^−^ cells, cultured them with IL‐2, and then co‐cultured them with HepG2, a HCC cell line, to mimic TME‐induced Ti‐Tregs (Figure S8A, Supporting Information). In this setting, we examined the effect of puerarin on human MIC19. CETSA showed that puerarin treatment reduced the thermal stability of MIC19 (Figure S8B, Supporting Information), whereas DARTS demonstrated increased protease sensitivity upon puerarin binding (Figure S8C, Supporting Information), indicating direct interaction. We then generated MIC19‐deficient nTregs via lentiviral shRNA knockdown (Figure S8D, Supporting Information). Puerarin significantly inhibited IL‐10 (Figure S8E, Supporting Information) and TGF‐β (Figure S8F, Supporting Information) secretion, downregulated ICOS (Figure S8G, Supporting Information) and CTLA‐4 (Figure S8H, Supporting Information) expression, and reduced Ti‐Treg proliferation (Figure S8I, Supporting Information); these inhibitory effects were abolished by MIC19 knockdown. Metabolically, puerarin markedly suppressed mitochondrial OXPHOS and ATP production (Figure S8J–L, Supporting Information) in Ti‐Tregs, which was also dependent on MIC19. In terms of suppressive function, puerarin‐treated Ti‐Tregs exhibited weakened inhibition of CD8⁺ T‐cell proliferation (Figure S8M, Supporting Information), and this effect was lost under MIC19 knockdown. Collectively, these results identify MIC19 as the critical molecular target through which puerarin modulates mitochondrial metabolism and suppressive function in Ti‐Tregs.

### 2.6 Puerarin Disrupts MIC19‐MIC60 Interaction and Promotes MIC19 Degradation in Tregs

**Figure 6:**
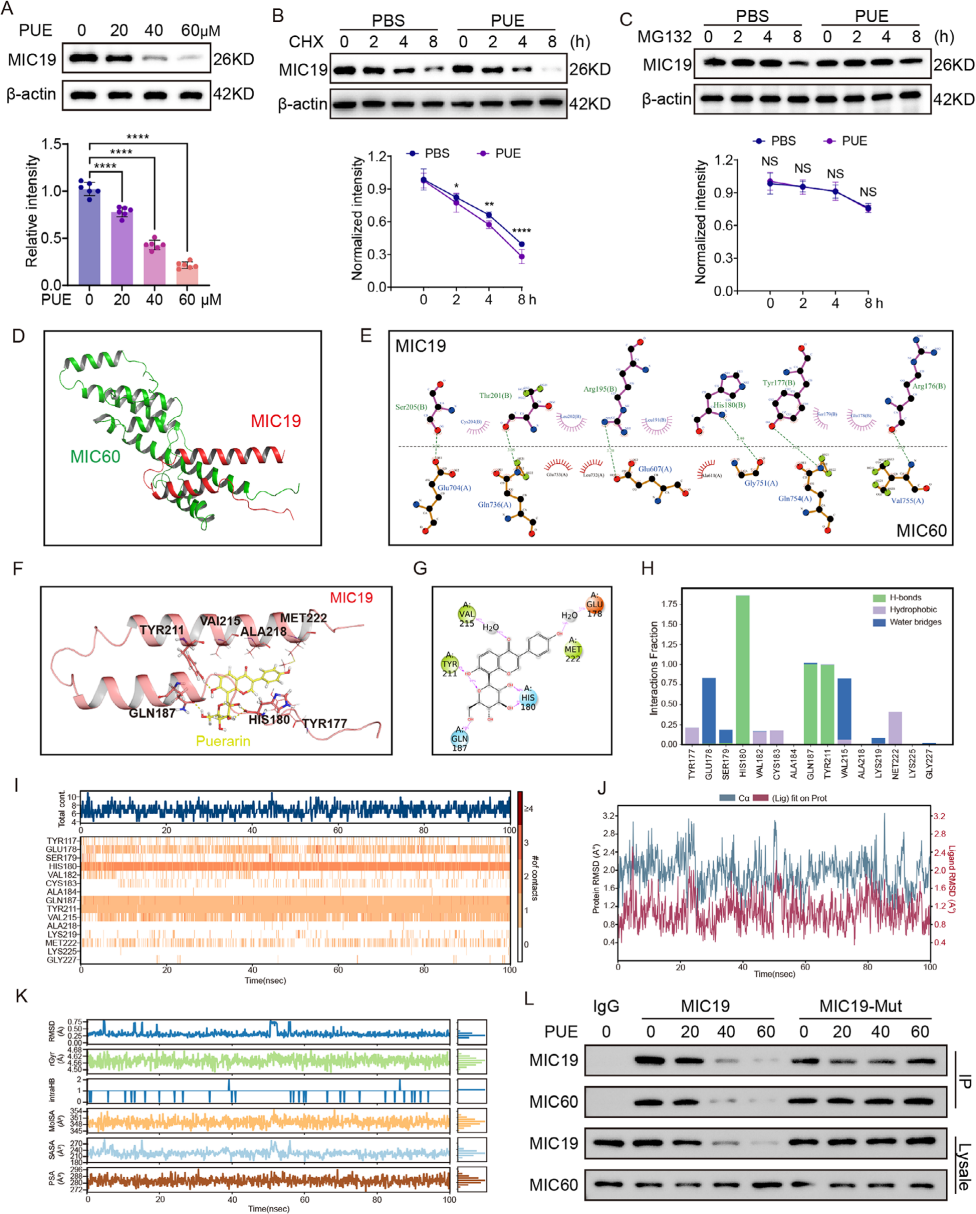
Puerarin (PUE) degrades MIC19 and regulates interaction with MIC60 in tumor‐infiltrating regulatory T cells (Ti‐Tregs). A) Western blot analysis of MIC19 protein levels in mice Ti‐Tregs treated with increasing concentrations of PUE (0, 20, 40, 60 µm) and co‐cultured with Hepa53.4 cells for 3 days (n = 3). B) Cycloheximide (CHX) chase assay of MIC19 protein stability in mice Ti‐Tregs pretreated with PBS or PUE (60 µm) and co‐cultured with Hepa53.4 cells for indicated durations (0–8 h), followed by western blotting (n = 3). C) Western blot analysis of MIC19 protein levels in mice Ti‐Tregs treated with PBS or PUE in the presence of MG132 (10 µm) for the indicated time points (0–8 h) (n = 3). D) Predicted structural model of the MIC19–MIC60 complex generated using AlphaFold3‐based protein–protein docking and visualized with PyMOL. E) LigPlot‐generated interface contact map showing predicted amino acid interactions between MIC19 and MIC60 derived from protein–protein docking simulation. F) Structural docking model showing the binding pocket of PUE on MIC19 near the MIC60 interface, visualized with PyMOL. G) 2D schema of the molecular interactions between PUE and key residues of MIC19, derived from docking analysis performed with the Schrödinger suite. H) Quantification of MIC19 residues contributing to PUE binding, categorized into hydrogen bonds, hydrophobic contacts, and water bridges. I) Molecular dynamics (MD) simulation showing root mean square fluctuation (RMSF) of MIC19 residues forming a complex with PUE during a 100 ns trajectory. J) Root mean square deviation (RMSD) and radius of gyration (Rg) curves of MIC19–PUE complex during MD simulation. (K) MD trajectory analyses of secondary structure transitions, hydrogen bond number, solvent‐accessible surface area (SASA), and structural compactness over 100 ns. L) Co‐immunoprecipitation (co‐IP) of MIC19 and MIC60 in mice Ti‐Tregs treated with increasing concentrations of PUE (0, 20, 40, 60 µm) and co‐cultured with Hepa53.4 cells, and in cells expressing MIC19‐binding‐deficient mutants. Total and co‐precipitated MIC19 and MIC60 were detected by western blot analysis. Data are presented as mean ± standard error of the mean (SEM). P‐values were calculated using one‐way analysis of variance (ANOVA) with Tukey's multiple comparisons in (A) and unpaired two‐tailed Student's *t*‐test in (B–C). ^*^ *P* < 0.05; ^**^ *P* < 0.01; ^***^ *P* < 0.001; ^****^ *P* < 0.0001.

To further elucidate the molecular mechanism by which puerarin regulates MIC19 in Ti‐Tregs, we conducted systematic analyses and observed that puerarin treatment led to a dose‐dependent reduction in MIC19 protein expression in Ti‐Tregs cultured with Hepa53.4 cells in vitro (Figure **6**A). Cycloheximide chase assays demonstrated that puerarin significantly shortened the half‐life of MIC19 under conditions of blocked protein synthesis (Figure 6B), indicating that puerarin promotes MIC19 degradation by accelerating its protein turnover.

Previous studies have established that the interaction between MIC19 and the scaffold protein MIC60 is structurally essential for the complex integrity of MICOS, maintaining mitochondrial cristae morphology and inner‐outer membrane contact sites.^[^ ^23^, ^24^ ^]^ Using AlphaFold3, we constructed a structural model of the MIC19–MIC60 complex (Figure 6C). Hydrogen bond analysis of the highest‐confidence conformation revealed that MIC19 residues Tyr177, His180, Arg195, and Ser205 form stable hydrogen bond networks with MIC60 residues Gln754, Gly751, Glu607, and Glu704, supporting complex stabilization (Figure 6D).

Subsequent molecular docking analysis showed that puerarin interacts with MIC19 via hydrogen bonds involving key residues His180, Gln187, Tyr211, and Val215 (Figure 6E, F), which lie within the critical interface region between MIC19 and MIC60. Molecular dynamics simulations further confirmed that puerarin forms high‐frequency hydrogen bonds with MIC19 at His180, Gln187, and Tyr211 (Figure 6G, H), suggesting stable and specific interactions at these sites.

The puerarin‐MIC19 complex also exhibited structural stability throughout the simulation, with root‐mean‐square deviation fluctuations consistently below 2 Å (Figure 6I). The radius of gyration remained within a narrow range (4.5–4.68 Å), indicating a compact ligand conformation. Solvent‐accessible, polar, and molecular surface areas showed minimal variations, suggesting that the complex did not undergo substantial conformational rearrangement and maintained stable polar interactions (Figure 6J).

Finally, co‐immunoprecipitation assays of Ti‐Tregs treated with increasing concentrations of puerarin and co‐cultured with Hepa53.4 cells demonstrated progressive reduction in MIC19 protein levels and decreased MIC60 co‐binding (Figure 6K). Together, these findings indicate that puerarin binds to key residues of MIC19—specifically His180, Gln187, and Tyr211—thereby promoting its degradation and competitively disrupting its interaction with MIC60. This dual mechanism destabilizes the MICOS complex, leading to mitochondrial dysfunction in Tregs.

### 2.7 Puerarin Suppresses Ti‐Treg Function via Direct Binding to MIC19 Residues His180, Gln187, and Tyr211

**Figure 7:**
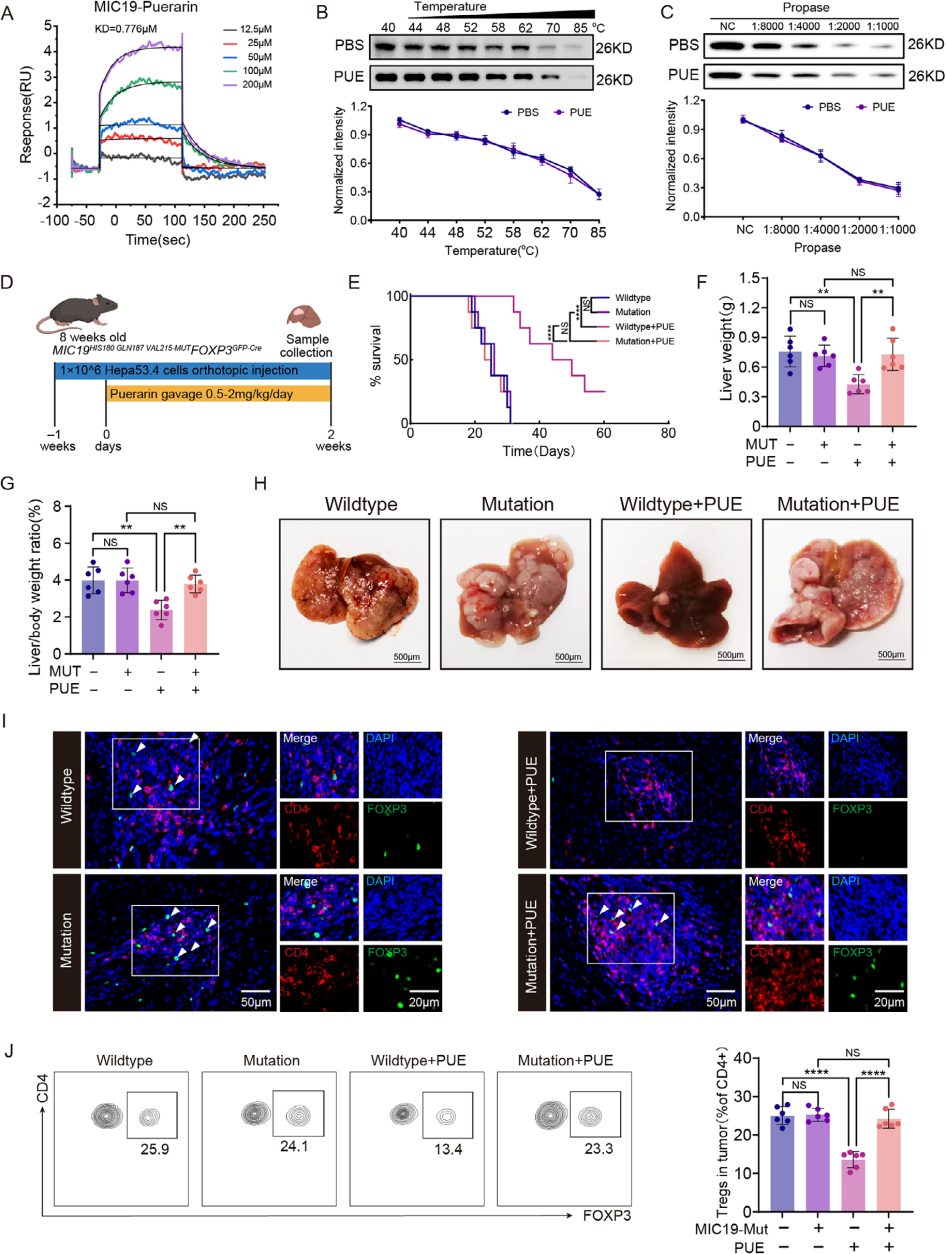
Mutation of MIC19 disrupts puerarin (PUE) binding. A) Binding affinity between MIC19 mutant (MIC19‐Mut) protein and PUE measured by surface plasmon resonance (SPR) at indicated concentrations (12.5–200 µm). B) Cellular thermal shift assay (CETSA) of MIC19‐Mut protein in mice tumor‐infiltrating regulatory T cells (Ti‐Tregs) treated with PBS or PUE (2.0 mg kg^−1^day^−1^) across a range of temperatures. Protein band intensity was normalized to 40 °C (n = 3). C) Drug affinity responsive target stability (DARTS) assay of MIC19‐Mut protein in mice Ti‐Tregs treated with PBS or PUE (2.0 mg kg^−1^day^−1^) and increasing concentrations of propase (n = 3). D) Experimental scheme: MIC19^HIS180/GLN187/VAL215‐Mut^FOXP3^GFP‐Cre^ mice orthotopically injected with 1 × 10⁶ Hepa53.4 cells and treated daily with oral gavage of puerarin (PUE) (0.5–2.0 mg kg^−1^day^−1^) starting one week post‐injection. E) Kaplan–Meier survival analysis of the MIC19‐Mut mice treated with PBS or PUE (n = 10). F) Liver weight of the MIC19‐Mut mice at experimental endpoint in each treatment group (n = 10) (Wildtype vs Mutation: HR = 1.00, 95% CI = 0.38–2.66; Wildtype vs Wildtype + PUE: hazard ratio [HR] = 0.53, 95% confidence interval [CI] = 0.018–0.153; Mutation vs Mutation + PUE: HR = 1.06, 95% CI = 0.40–2.81; Wildtype + PUE vs Mutation + PUE: HR = 2.52, 95% CI = 0.088–0.727). G) The liver‐to‐body weight ratio of the MIC19‐Mut mice in each treatment group (n = 10). H) Representative image of hepatocellular carcinoma (HCC) from 3 weeks after treatment of MIC19‐Mut mice with different doses of PUE. I) Immunofluorescence staining of the tissues obtained from MIC19‐Mut mice showing CD4⁺FOXP3⁺ Treg infiltration in HCC model mice treated with PBS or PUE (2.0mg/kg/day) (n = 3). Scale bars: overview = 50 µm; inset = 20 µm. J) Flow cytometry analysis of Ti‐Tregs in MIC19‐Mut mice treated with PBS or PUE (2.0 mg kg^−1^day^−1^) (n = 6). Data are presented as mean ± standard error of the mean (SEM). P‐values were calculated using a log‐rank test for survival analysis in (E) and one‐way analysis of variance (ANOVA) with Tukey's multiple comparisons in (F–G) and (J). ^*^ *P* < 0.05; ^**^ *P* < 0.01; ^***^ *P* < 0.001; ^****^ *P* < 0.0001.

Building on the preceding computational analyses, we generated a mutant MIC19 protein in which three key residues—His180, Gln187, and Tyr211—were substituted with alanine and subsequently purified the recombinant protein. Surface plasmon resonance analysis revealed that the binding affinity of puerarin to the mutant MIC19 was markedly reduced compared with the wild type (KD = 0.776 µm vs 14.26 µm), representing a 54.4 fold decrease in binding strength (Figure **7**A). CETSA and DARTS assays further confirmed that these mutations altered the biophysical properties of MIC19, abolishing puerarin‐induced thermal destabilization (Figure 7B) and protease sensitivity enhancement (Figure 7C).

To assess the in vivo relevance of these interaction sites, we generated a genetically modified mouse strain expressing Treg‐specific point mutations in MIC19 (His180, Gln187, and Tyr211). Primary Tregs were isolated from these mice and co‐cultured with Hepa53.4 cells. Immunoprecipitation experiments demonstrated that puerarin failed to induce MIC19 degradation or disrupt the MIC19–MIC60 complex (Figure 6K), underscoring the critical role of these residues in puerarin‐mediated effects.

In the corresponding mutant murine HCC model mice (Figure 7D), puerarin treatment conferred no survival benefit (Figure 7E), nor did it reduce tumor burden (Figure 7F–H) or suppress Ti‐Treg infiltration (Figure 7I, J), indicating that MIC19 mutation abrogates the therapeutic efficacy of puerarin in vivo. Consistently, CD4⁺ naïve T cells isolated from these mutant mice and co‐cultured with Hepa53.4 cells showed complete resistance to puerarin‐mediated inhibition of Ti‐Treg differentiation and IL‐10/TGF‐β production of Ti‐Tregs (Figure S9A, B, Supporting Information). In CD8⁺ T cell co‐culture assays, puerarin also failed to attenuate the immunosuppressive function of mutant Ti‐Tregs (Figure S9C, Supporting Information).

At the metabolic level, both OCR and ATP production remained unaffected by puerarin treatment in mutant Ti‐Tregs (Figure S9D, E, Supporting Information). TEM, JC‐1 staining, MitoSOX, and apoptosis assays similarly revealed no mitochondrial dysfunction following puerarin exposure (Figure S9F–J, Supporting Information). Collectively, these results provide compelling genetic evidence that puerarin primarily exerts its immunometabolic effects on Ti‐Tregs by binding to the critical residues His180, Gln187, and Tyr211 of MIC19.

## 3 Discussion

In this study, we identified puerarin as a first‐in‐class, natural immunometabolic modulator that selectively disrupts Ti‐Treg function through mitochondrial targeting. Puerarin preferentially accumulates in Ti‐Tregs, impairing their suppressive function and enhancing CD8⁺T cell‐mediated antitumor immunity. Mechanistically, puerarin binds to key residues (His180, Gln187, Tyr211) of MIC19, disrupting its interaction with MIC60 and destabilizing the MICOS complex, which leads to cristae disorganization and OXPHOS collapse. This mitochondrial perturbation triggers metabolic paralysis in Tregs, attenuating their immunosuppressive capacity while sparing effector immunity. The loss of puerarin efficacy in MIC19‐mutant models further highlights the essential role of the MIC19‐MIC60 axis in mediating its immunomodulatory function. Our findings reveal a druggable link between mitochondrial architecture and Treg metabolism and provide a mechanistic basis for developing phytochemical‐based precision immunotherapies against HCC.

The selective activity of puerarin toward Ti‐Tregs is attributed to two key features: its pharmacokinetic enrichment in the liver and its mechanistic targeting of OXPHOS‐dependent cells. First, in vivo imaging and organ quantification showed that puerarin preferentially accumulates in the liver following oral administration, where it can readily access tumor‐infiltrating immune cells, such as Ti‐Tregs. Second, although puerarin is a non‐targeted natural compound, it binds with high specificity to MIC19—a mitochondrial inner membrane protein critical for cristae integrity and OXPHOS maintenance.^[^ ^22^, ^25^ ^]^ Ti‐Tregs rely heavily on mitochondrial respiration rather than glycolysis to sustain their suppressive phenotype in the hostile TME.^[^ ^13^, ^26^ ^]^ Our single‐cell transcriptomic analysis of human HCC confirmed that among tumor‐infiltrating lymphocytes, Ti‐Tregs exhibit the highest enrichment of OXPHOS‐related gene signatures. Thus, the combined effect of local accumulation and metabolic vulnerability renders Ti‐Tregs susceptible to puerarin‐mediated immunometabolic disruption.

OXPHOS plays a central role in sustaining the stability and suppressive function of the Ti‐Treg lineage.^[^ ^27^, ^28^ ^]^ Unlike effector T cells, which rely on glycolysis,^[^ ^14^, ^29^ ^]^ Ti‐Tregs depend on mitochondrial respiration to support fatty acid oxidation and maintain FOXP3 expression. Disruption of OXPHOS impairs Ti‐Treg viability, reduces IL‐10 production, and destabilizes lineage‐defining transcriptional programs.^[^ ^30^, ^31^ ^]^ Thus, mitochondrial metabolism is integral to Ti‐Treg identity. By targeting MIC19—a key structural component of the MICOS complex—puerarin disrupts cristae architecture, leading to OXPHOS collapse and selective metabolic disruption of Ti‐Tregs.

The MICOS complex is a conserved multi‐protein assembly essential for maintaining cristae junctions and mitochondrial inner membrane architecture.^[^ ^32^, ^33^ ^]^ MIC60 acts as the core scaffold, while MIC19 bridges MIC60 to outer membrane proteins, such as Sam50, ensuring intermembrane contact site stability.^[^ ^22^, ^24^, ^34^ ^]^ Disruption of the MIC19–MIC60 interaction leads to cristae disorganization, impaired supercomplex assembly, and defective OXPHOS. These structural perturbations may compromise mitochondrial respiration in Ti‐Tregs, thereby impacting their metabolic and immunosuppressive function.

Our findings propose a previously unrecognized strategy: targeting cristae‐resident proteins to selectively disrupt Ti‐Treg metabolism. While prior efforts have aimed to suppress Ti‐Treg activity by inhibiting glycolysis, fatty acid oxidation, or amino acid metabolism, such approaches often lack specificity and may compromise effector T cell function.^[^ ^35^, ^36^, ^37^, ^38^ ^]^ In contrast, puerarin achieves Ti‐Treg‐selective inhibition by engaging a structural mitochondrial target, leaving other immune subsets largely unaffected. Although proteins such as OPA1 and Drp1 have been implicated in Treg metabolic regulation, this study is the first to position MIC19 as a functional nexus between mitochondrial ultrastructure and Treg immunosuppression.^[^ ^39^, ^40^, ^41^ ^]^

Despite these advances, several limitations remain. First, the broader effects of puerarin on other immunosuppressive populations, such as myeloid‐derived suppressor cells, tumor‐associated macrophages, and dendritic cells, have not been fully explored. Second, while in vivo studies support its antitumor activity, the pharmacokinetics, long‐term safety, and tissue specificity of puerarin require further evaluation, particularly given the ubiquitous expression of MIC19 in metabolically active non‐immune cells.^[^ ^22^, ^25^ ^]^ Third, validation in human Treg systems is still lacking, and cross‐species conservation of the MIC19‐dependent mechanism needs to be confirmed.

Future work may benefit from multi‐omics approaches, including single‐cell and spatial transcriptomics and metabolomics, to characterize the full spectrum of puerarin's immunomodulatory effects. Pharmacokinetic and bioengineering strategies, such as nanoparticle encapsulation or prodrug design, may improve its systemic stability and bioavailability.^[^ ^42^, ^43^ ^]^ Conditional deletion models and organ‐specific metabolic profiling will help delineate off‐target risks. Crucially, validating the MIC19‐dependent effects in human Tregs through gene editing and functional assays would support translational potential.

This study also opens new research directions. Other MICOS components, such as MIC60 or MIC10, may hold unexplored roles in immune regulation. The upstream signals governing MIC19 stability, such as oxidative stress, nutrient status, or post‐translational modifications, may provide additional therapeutic targets. Finally, structure‐based optimization of puerarin could yield high‐affinity derivatives or enable the development of MIC19‐targeting PROTACs or molecular glue degraders. Given the central role of Tregs across cancer, autoimmunity, and transplantation, targeting mitochondrial architecture represents a promising avenue for next‐generation immunotherapies.

## 4 Experimental Section

### Ethical Approval Statement

This study was approved by the Ethics Committee of the First Affiliated Hospital with Nanjing Medical University (2024‐SRF A‐068), and written informed consent was obtained from all patients in accordance with the Declaration of Helsinki. Animal experiments were approved by the Institutional Animal Care and Use Committee of Nanjing Medical University (IACUC‐2310108) and performed in accordance with the relevant guidelines and regulations.

### Mice

Wild‐type (WT) C57BL/6J mice were purchased from the Animal Core Facility of Nanjing Medical University (Nanjing, China), and originally obtained from the Jackson Laboratory. Foxp3‐GFP mice (C57BL/6J) were donated by Professor Zhexiong Lian, Guangdong Medical Academy, China. Foxp3^GFP‐Cre^ mice (C57BL/6J) were donated by Professor Bin Li, Shanghai Jiao Tong University, China. Rag1^fl/fl^ mice were purchased from GemPharmatech (Nanjing, China), MIC19^His180, Gln187, Tyr211^Foxp3^YFP‐cre^ were generated by were generated by Shanghai Model Organisms Center. Foxp3^DTR^ mice were purchased from the Shanghai Model Organisms Center. All animal experiments were approved by the Laboratory Animal Welfare Ethics Committee (IACUC) of Nanjing Medical University (Approval No. IACUC‐2310108). Age‐matched (6–10 weeks) and gender‐matched (male) mice were used. Mice were housed in a controlled environment with temperature (22 ± 1 °C) and humidity (60% ± 10%) under specific pathogen‐free conditions, with a 12‐h light‐dark cycle.

### Murine Tumor Models

Hepa53.4 cells (1 × 10⁶ cells in 100 µL) were orthotopically injected into the liver of recipient mice. Mice were sacrificed and analyzed on day 21 post‐injection. Puerarin was administered daily via oral gavage at doses ranging from 0.5 to 2.0 mg kg^−1^day^−1^, starting one week after tumor implantation and continuing for 14 consecutive days. Foxp3^DTR^ mice received intraperitoneal diphtheria toxin (DT) at 25 ng g^−1^ every other day to selectively deplete regulatory T cells. Mice exhibiting signs of excessive tumor burden (≥10% of body weight), severe ascites, prostration, lethargy, unresponsiveness, or failure to groom were considered to have reached humane endpoints and were recorded as deceased.

### In Vivo Biodistribution of PUE‐CY7

To assess the in vivo biodistribution of puerarin, a near‐infrared fluorescent probe, PUE‐CY7, was synthesized by chemically conjugating puerarin with the fluorescent dye Cyanine7 (CY7). Female C57BL/6 mice bearing orthotopic Hepa1‐6 tumors were orally gavaged with PUE‐CY7 (2 mg kg^−1^) dissolved in 0.5% carboxymethyl cellulose sodium (CMC‐Na). At various time points post‐gavage (0, 1, 2, and 3 h), mice were anesthetized with isoflurane and subjected to in vivo imaging using an IVIS Spectrum system (PerkinElmer, USA) with the excitation/emission wavelength set at 745/800 nm.

After the final imaging, mice were euthanized, and major organs, including the tumor, liver, spleen, kidney, lung, and heart, were harvested for ex vivo imaging to assess tissue distribution. Fluorescence intensity was quantified using Living Image software (PerkinElmer, USA) and normalized against the background signal.

### Immunofluorescence and Mitochondrial Membrane Potential Analysis

Tissue samples were dehydrated, cleared, and embedded in paraffin using a tissue processor. Sections were cut, baked, and subjected to antigen retrieval followed by serum blocking. For cellular staining, cells were collected, washed three times with PBS, fixed in 4% paraformaldehyde on glass slides, permeabilized with 0.1% Triton X‐100, and blocked with 5% BSA. Primary antibodies were applied and incubated overnight at 4 °C. After washing, slides were incubated with appropriate fluorescent secondary antibodies for 60 min at room temperature. Nuclear staining was performed using Hoechst dye. Mitochondrial membrane potential was assessed using the JC‐1 Mitochondrial Membrane Potential Assay Kit (MCE, HY‐K0601) according to the manufacturer's instructions. Fluorescent images were acquired and analyzed using ImageJ software (version 1.8.0).

### Western Blotting

Collected cells were washed with PBS and lysed using radioimmunoprecipitation assay (RIPA) buffer supplemented with phosphatase and protease inhibitors (Beyotime, China). Protein concentrations were determined using the BCA Protein Assay Kit (Beyotime). Equal amounts of protein were separated by SDS‐PAGE and transferred onto PVDF membranes. Membranes were blocked and incubated with primary antibodies, followed by incubation with appropriate secondary antibodies. Protein bands were visualized using Image Lab software (version 5.2.1). The following primary antibodies were used: anti‐β‐Actin (Abcam, ab8226), anti‐MIC19 (Abcam, ab224565), anti‐MIC60 (Abcam, ab110329), and Total OXPHOS Rodent WB Antibody Cocktail (Abcam, ab110413).

### Mice T Cell Isolation

Murine leukocyte suspensions were prepared from the tumor or spleen. CD4⁺CD25⁺ regulatory T cells (Tregs) and CD8⁺ T cells were isolated using the auto‐MACS (Miltenyi Biotec, San Diego, CA, USA) based on surface marker expression. Purified cells were cultured in RPMI 1640 medium supplemented with 10% heat‐inactivated fetal bovine serum (FBS), 1% penicillin‐streptomycin, 1% HEPES buffer, and 0.1% 2‐mercaptoethanol. Recombinant IL‐2 (10 ng mL^−1^; R&D Systems, Minneapolis, MN, USA) was added every 2–3 days according to the degree of T cell expansion. Anti‐CD3/CD28 mAb‐coated activation beads (Thermo Fisher Scientific, Massachusetts, USA; Cat. No. 11453D) were added at the start of each stimulation at a bead‐to‐cell ratio of 1:1.

### Human T Cell Isolation

Peripheral blood mononuclear cells (PBMCs) were isolated from leukapheresis products of healthy volunteers at Nanjing Medical University using Ficoll‐Hypaque density gradient centrifugation (Amersham Biosciences, Slough, Buckinghamshire, UK). Regulatory T cells (Tregs) were subsequently enriched using an autoMACS system (Miltenyi Biotec, San Diego, CA, USA) based on the surface markers CD4⁺CD25⁺CD127^−^, while CD8⁺ T cells were isolated based on CD8⁺ expression. Tregs and CD8⁺ T cells were cultured in X‐VIVO 15 medium (BioWhittaker, Walkersville, USA) supplemented with 10% human AB serum (Valley Biomedical, Winchester, VA, USA). Recombinant human IL‐2 (10 ng mL^−1^; R&D Systems, Minneapolis, MN, USA) was added every 2–3 days depending on the proliferation status of T cells. Cells were activated using anti‐CD3/CD28 monoclonal antibody‐coated beads (Thermo Scientific, Massachusetts, USA; Cat. No. 11132D) at a bead‐to‐cell ratio of 1:1 at the initiation of each stimulation.

### Co‐Culture of Tregs and Hepa53.4 Cells

Naïve CD4⁺ T cells were isolated from the spleens of C57BL/6 mice and resuspended at a density of 1 × 10⁶ cells per well in a 24‐well plate. Cells were activated using anti‐CD3/CD28 activation beads (bead‐to‐cell ratio 1:1) in the presence of 100 U mL^−1^ IL‐2. On the day prior to co‐culture, Hepa53.4 tumor cells were seeded in the lower chamber of the wells. The tregs were then co‐cultured with Hepa53.4 cells for 3–5 days under Treg‐polarizing conditions. After co‐culture, Tregs were harvested, washed with PBS, and subjected to flow cytometry analysis.

### Suppression Assay

CD8⁺ T cells were isolated using the auto‐MACS (Miltenyi Biotec, San Diego, CA, USA) based on CD8⁺ surface expression and subsequently labeled with CFSE (Invitrogen, Carlsbad, CA, USA). Labeled cells were stimulated with anti‐CD3/CD28‐coated beads at a 1:1 bead‐to‐cell ratio. Prior to co‐culture, Tregs were pretreated either with or without Puerarin (PUE, 60 µm) for 3 days. For the suppression assay, CD8⁺ T cells (responders) were co‐cultured with untreated or PUE‐pretreated Tregs at varying ratios (Treg: CD8⁺ ratios of 1:1, 1:4), in the presence of anti‐CD3/CD28 stimulation. On day 4, cells were stained with anti‐CD4 and anti‐CD8 antibodies, and T cell proliferation was assessed by CFSE dilution. Suppressive capacity was quantified using the Division Index in FlowJo software (v10.8.1).

### Flow Cytometry

Tumor tissues were processed into single‐cell suspensions as follows: excised tumor samples were finely minced with sterile scissors and digested in a collagenase solution at 37 °C for 1 h in a water bath. The resulting cell suspensions were centrifuged at 50 × *g* for 5 min at 4 °C to remove parenchymal cells. Immune cells were then enriched by collecting the “mist‐like” interphase after density gradient centrifugation using 25% Percoll at 2300 rpm for 23 min.

Isolated cells were incubated with the indicated surface antibodies for 30 min at room temperature. Prior to staining, Fc receptors were blocked with an anti‐mouse CD16/32 antibody (BD Pharmingen, 553142) for 20 min. Cells were then stained with antibodies targeting CD45 (BV711, BD Horizon, 563709), CD3 (BUV737, BD Horizon, 741716), CD4 (BUV395, BD Pharmingen, 563790), CD8 (Brilliant Violet 650 BioLegend, 100742), ICOS (APC, BioLegend, 313509), and CTLA‐4 (Brilliant Violet 421, BioLegend, 106311).

For mitochondrial membrane potential assessment, cells were stained using the JC‐1 Mitochondrial Membrane Potential Assay Kit (MCE, HY‐K0601) following the manufacturer's protocol. Data acquisition was performed on a FACSCelesta flow cytometer (BD Biosciences, New Jersey, USA), and data analysis was conducted using FlowJo software (v10.8.1, Beckman Coulter, USA).

### Mitochondrial Function and Metabolic Analysis

To assess mitochondrial membrane potential, Treg cells were incubated at 37 °C for 30 min with 200 nM TMRE (Abcam), 100 nM MitoTracker Red (Thermo Fisher Scientific, M22425), and 1 µm MitoSOX Green (Thermo Fisher Scientific, M36005) according to the manufacturer's protocols. Fluorescence signals were visualized and quantified using ImageJ software (v1.8.0).

Treg oxidative phosphorylation (OXPHOS) was evaluated using a Seahorse XF96 Extracellular Flux Analyzer (Agilent Technologies). For oxygen consumption rate (OCR) measurements, mouse Tregs were sequentially treated with 1.5 µm oligomycin, 1.0 µm FCCP, 1 µm rotenone, and 1.8 µm antimycin A (Agilent Mitostress Kit). OCR data were recorded and analyzed using Seahorse Wave software.

### ATP Content Measurement

ATP levels were quantified using the Enhanced ATP Assay Kit (Beyotime, S0027) following the manufacturer's protocol. Briefly, cells from each experimental group were lysed with 100 µL of the provided lysis buffer and centrifuged at 12,000 × *g* for 20 min at 4 °C. Subsequently, 10 µL of the resulting supernatant was mixed with 100 µL of ATP detection solution. Luminescence intensity was measured using a luminometer (Varioskan Flash, Thermo Fisher Scientific). ATP concentrations were determined by reference to a standard curve and normalized to protein concentration in the corresponding supernatants.

### Mitochondrial Electron Microscopy Detection

Samples were initially fixed with an electron microscopy fixative, followed by post‐fixation in 1% osmium tetroxide. Dehydration was performed through a graded acetone series (30% to 100%). The specimens were then infiltrated and embedded in Epon‐812 resin using a series of transitional mixtures (acetone: Epon‐812 ratios of 3:1, 1:1, and 1:3). Ultrathin sections (60–90 nm) were obtained using an ultramicrotome and mounted onto copper grids. The sections were stained with uranyl acetate at room temperature for 10–15 min, followed by lead citrate staining for 1–2 min. Images were acquired using a JEM‐1400FLASH transmission electron microscope (JEOL). Mitochondrial injuries were assessed using the Flameng score. Five microscopic fields were randomly selected to obtain the mean Flameng score for each group and the mitochondria were graded according to the following criteria: Grade 0 (score 0), mitochondria with normal ultramicrostructure and intact granules; Grade I (score 1), mitochondria with basically normal ultramicrostructure and partial loss of granules; Grade II (score 2), swollen mitochondria with transparent matrices; Grade III (score 3), mitochondria with transparent matrices and fragmented cristae or formation of flocculent densities in their mitochondrial matrices; and Grade IV (score 4), mitochondria lacking matrix with fragmented cristae and disrupted outer membranes.

### RNA Extraction and Quantitative Real‐Time PCR (RT‐qPCR)

Total cellular RNA was extracted using TRIzol reagent (15596018, Invitrogen), and RNA concentrations were quantified with a NanoDrop spectrophotometer (Thermo Fisher Scientific). Reverse transcription was performed using a cDNA synthesis kit (R323‐01, Vazyme, China). Quantitative real‐time PCR was conducted using the ChamQ Universal SYBR qPCR Master Mix (Q711‐02, Vazyme, China) according to the manufacturer's protocol. The following primers were used for the detection of MIC19:

**Table:** 

Gene	Forward (5’ to 3’ sequence)	Reverse (5’ to 3’ sequence)	Species
**MIC19**	AGTCCTCTCCATCTGGCTCTAAGTC	CGCATCACTCGGTCTTTCTCTTCC	Mouse
**MIC19**	CGAAGTCTCAGCGGTATTCTGG	TCTCGGTCCAGCTCTTTGGCTT	Human

### RNA Sequencing (RNA‐Seq) Analysis

Mouse Treg cells were stimulated with 60 µm PUE for 24 to 48 h, after which total RNA was extracted using TRIzol reagent (Invitrogen, CA, USA). RNA purity and concentration were assessed using a NanoDrop 2000 spectrophotometer (Thermo Scientific, USA), and RNA integrity was evaluated with the Agilent 2100 Bioanalyzer (Agilent Technologies, Santa Clara, CA, USA). cDNA libraries were constructed using the VAHTS Universal V6 RNA‐seq Library Preparation Kit, following the manufacturer's instructions. Transcriptome sequencing and bioinformatic analyses were conducted by OE Biotech Co., Ltd. (Shanghai, China). Differentially expressed genes (DEGs) were identified with a threshold of Q‐value < 0.05 and fold change > 2 or < 0.5. KEGG pathway enrichment analysis of DEGs was performed in R (v3.2.0) based on a hypergeometric distribution model, and significantly enriched pathways were identified.

### Lentivirus Transduction

Tregs were isolated as described above. Following activation with IL‐2 (10 ng mL^−1^) for 24 h, cells were transduced with retrovirus carrying plvx‐U6‐shMIC19‐EGFP for gene knockdown, or the corresponding overexpression construct. Retroviral supernatants were supplemented with polybrene (4 µg Ml^−1^) and centrifuged onto the cells at 450 g for 2 h. Post‐transduction, Tregs were cultured at 37 °C in a humidified incubator with 5% CO_2_. For downstream experiments, EGFP⁺ Tregs were sorted 48 h after transduction and maintained in a complete medium for an additional 48 to 72 h.

### Fluorescence Labeling of PUE‐DA in Tregs

Tregs (1 × 10⁶) were cultured in 6‐well plates for 3 days and incubated with varying concentrations of PUE‐DA (0–100 µm) for 1 hour. After drug exposure, the culture medium was discarded, and cells were irradiated with UV on ice for 15 min. Following EDTA‐trypsin treatment, cells were collected by centrifugation at 1500 × g for 5 min. The pellets were washed, resuspended in 100 µL PBS, and lysed via sonication (25% amplitude, 2 s on/2 s off, total 2 min). The lysates were clarified by centrifugation at 14 000 × g for 30 min at 4 °C.

Click chemistry was performed using a copper‐catalyzed azide‐alkyne cycloaddition (CuAAC) reaction. The click mix was freshly prepared with 0.2 mm azide‐TAMRA (from 20 mm stock in DMSO), 0.1 mM TBTA (from 10 mm DMSO stock), 1.0 mm TCEP (from 100 mm ddH_2_O stock), and 1.0 mm CuSO_4_ (from 100 mm ddH_2_O stock), adjusted to 200 µL final volume. Samples were gently mixed and incubated at 37 °C for 1 h with shaking at 400 rpm.

Proteins were precipitated by adding 800 µL of ice‐cold acetone, washed twice with chilled methanol, air‐dried, and resuspended in 40 µL of 0.4% SDS in PBS along with 10 µL of 5× SDS loading buffer. After denaturation at 95 °C for 10 min and brief cooling on ice, proteins were resolved by 10% SDS‐PAGE. Fluorescence signals were detected using a Typhoon FLA 9500 scanner (GE Healthcare). Total proteins were visualized by Coomassie Brilliant Blue staining.

For competition assays, cells were pretreated with a 20 fold molar excess of free puerarin for 30 min prior to PUE‐DA incubation. DMSO served as the vehicle control in all treatment groups.

### Surface Plasmon Resonance and Amino Acid Mutation

The interaction between MIC19 and puerarin was evaluated using an OpenSPR instrument (Nicoya, Canada). A carboxyl sensor chip (SEN‐AU‐100‐3‐COOH; Nicoya, Canada) was used to immobilize recombinant MIC19 protein via standard amine coupling chemistry. Puerarin solutions at various concentrations (12.5–200 µm) were prepared in a running buffer and injected at a constant flow rate of 20 µL min^−1^. The dissociation constant (K_D) was calculated using TraceDrawer software based on a 1:1 Langmuir binding model.

To identify critical residues mediating puerarin‐induced stabilization of MIC19, alanine scanning mutagenesis was performed using the Residue Scanning module in Schrödinger (version 2021‐4). Selected amino acid residues were individually substituted with alanine, and changes in binding free energy (ΔG<sub>bind</sub>) were calculated relative to the wild‐type structure to evaluate the contribution of each residue to binding affinity.

### Cellular Thermal Shift Assay (CETSA)

Cell lysates were evenly divided into two parts. One was treated with vehicle as a negative control, and the other was incubated with puerarin at room temperature for 1 h to allow compound–protein interaction. After incubation, both sets of lysates were exposed to a series of defined temperatures ranging from 40 to 85 °C for 5 min to induce thermal denaturation. The samples were then returned to room temperature to allow stabilization. Subsequently, the lysates were centrifuged at 12 000 rpm for 10 min at 4 °C to separate the soluble protein fractions. The supernatants were carefully collected and analyzed via Western blotting to determine changes in protein thermal stability following puerarin treatment.

### Drug Affinity Responsive Target Stability (DARTS)

Cell lysates were first incubated with puerarin at room temperature for 30 min to facilitate potential compound–protein interactions. Following this preincubation, Propase was added at various enzyme‐to‐protein ratios ranging from 1:1,000 to 1:8,000. The mixtures were then incubated at 40 °C for an additional 30 min to allow proteolytic digestion. To terminate the enzymatic reactions, SDS loading buffer was added directly to the samples. The resulting mixtures were analyzed by SDS‐PAGE, and immunoblotting was performed to evaluate the relative stability of the target proteins under proteolytic stress.

### Cell Migration Test

The migratory capacity of Hep53.4 cells was assessed using both scratch wound and Transwell assays. Cells were treated with varying concentrations of puerarin (PUE; 0, 50, 100, 150, and 200 µM; MedChemExpress, HY‐N0145). For the scratch assay, when cells reached ≈90% confluence, a linear wound was created using a sterile 1 mL pipette tip. To minimize proliferation effects, mitomycin C was added to the culture medium to minimize the effects of cell proliferation. Cells were then incubated under specified conditions, and images were acquired at 0, 12, and 24 h using an optical microscope (ZEISS, Germany). Wound closure was quantified using ImageJ software (NIH, USA).

For the Transwell migration assay, 100 µL of serum‐free cell suspension (1 × 10⁶ cells mL^−1^) was seeded into the upper chamber of a Transwell insert, while the lower chamber contained medium with the corresponding PUE treatments. After 24 h, migrated cells on the lower membrane surface were fixed with 4% paraformaldehyde and stained with 0.1% crystal violet (Biosharp, BLB802A). Images were captured using a light microscope (SOPTOP, China), and quantification was performed using ImageJ software.

### Cell Cloning and Proliferation

Cell proliferation was evaluated using the Cell Counting Kit‐8 (CCK‐8; Biosharp, BS350B) according to the manufacturer's instructions. Briefly, Hepa53.4 cells (5 × 10⁴) were suspended in a treatment medium and seeded into 96‐well plates. At the indicated time points, 100 µL of CCK‐8 working solution was added to each well, followed by incubation at 37 °C for 2 h. The absorbance was measured at 450 nm using a microplate reader (BioTek, CA, USA).

### Enzyme‐Linked Immunosorbent Assay (ELISA)

The levels of relevant cytokines, including TGF‐β and IL‐10, were quantified using enzyme‐linked immunosorbent assay (ELISA) kits (Jingmei Bio, Jiangsu, China) following the manufacturer's instructions. Absorbance values were measured using a microplate reader (BioTek, CA, USA), and cytokine concentrations were calculated based on the corresponding standard curves.

### LC‐MS/MS Analysis

Liquid chromatography‐tandem mass spectrometry (LC‐MS/MS) analysis was carried out using a Q Exactive HF or HFX mass spectrometer (Thermo Scientific) coupled to an Easy‐nLC system (Proxeon, Thermo Fisher Scientific). Peptide samples were first loaded onto a reverse‐phase trap column (Acclaim PepMap100, 100 µm × 2 cm, C18, Thermo Scientific), and then separated on an analytical C18 column (Easy Column, 75 µm × 10 cm, 3 µm particle size) using a linear gradient of buffer B (84% acetonitrile with 0.1% formic acid) in buffer A (0.1% formic acid) at a constant flow rate of 300 nL/min under IntelliFlow regulation.

The mass spectrometer was operated in positive ion mode with a data‐dependent acquisition strategy (top‐10 method). Full MS scans were acquired over an m/z range of 300–1800 at a resolution of 70 000 (at m/z 200). The top 10 most intense precursor ions were selected for higher‐energy collisional dissociation (HCD) with a normalized collision energy of 30 eV and an isolation window of 2 m/z. Fragment ion spectra were acquired at a resolution of 17 500. The AGC target was set to 3e6, with a maximum injection time of 10 ms. Dynamic exclusion was applied with a 40 s window, and peptide recognition mode was enabled. An underfill ratio of 0.1% was used to improve the sampling of low‐abundance ions.

### Molecular Docking Analysis

We constructed the MIC60–MIC19 complex using AlphaFold3 and selected the MIC60–MIC19 structure with the highest confidence score. Protein‐protein interactions between MIC60 and MIC19 were analyzed using LigPlot. The molecular docking of MIC19 with puerarin was performed using Schrödinger software (version 2021‐4). Briefly, the Protein Preparation Wizard was used to prepare the protein‐ligand complex, with energy minimization conducted under the OPLS2005 force field and protonation states assigned at pH 7.0 ± 2.0. The 3D protein structure was preprocessed in Maestro, including bond correction, residue completion, addition of hydrogen atoms, and removal of water molecules and non‐ligand heteroatoms. PyMOL was used for structural visualization. To verify the stabilizing effect of puerarin on CHCHD3, Desmond software (version 2021) was used to perform a 100‐ns molecular dynamics (MD) simulation of the MIC19–puerarin complex. The OPLS2005 force field was applied to parameterize both the protein receptor and the ligand. The protein‐ligand complex was solvated using the SPC water model, and Na⁺ and Cl^−^ ions were added to mimic physiological conditions. Periodic boundary conditions were applied using an orthorhombic box, and isothermal–isobaric (NPT) ensemble relaxation was performed to ensure system stability, with temperature maintained at 300 K and pressure at 1 atm. The 100‐ns MD simulation was followed by calculations of root mean square deviation (RMSD), root mean square fluctuation (RMSF), radius of gyration (Rg), and hydrogen bond analysis between the protein and ligand.

### Single‐Cell RNA Sequencing Data Analysis

Single‐cell RNA sequencing (scRNA‐seq) data used in this study were obtained from the GEO database (https://www.ncbi.nlm.nih.gov/geo/; accession number GSE162616). Four hepatocellular carcinoma (HCC) tumor samples, three healthy liver samples, and one HCC tumor‐adjacent sample were selected for subsequent analyses. Data processing and analyses were performed in the R environment (v4.2.0), primarily using the Seurat package (v4.0.0) for quality control, normalization, dimensionality reduction, clustering, and visualization. For each sample, quality control was conducted based on dataset‐specific sequencing characteristics, including the number of detected genes per cell (nFeature_RNA), UMI counts (nCount_RNA), and the proportion of mitochondrial genes (percent.mt), to remove low‐quality cells and potential doublets. The filtered data were then normalized using the LogNormalize method. Highly variable genes were identified using the FindVariableFeatures function and used for downstream dimensionality reduction. To integrate multiple samples and correct for batch effects, the Harmony algorithm was applied. Principal component analysis (PCA) was performed for linear dimensionality reduction, and UMAP was used for visualization. Cell type annotation was conducted manually based on canonical marker genes, supplemented by literature and database references. Expression of marker genes and gene sets of interest was visualized using FeaturePlot, VlnPlot (violin plots), and DotPlot functions. Pathway activity was assessed using the Hallmark gene sets from the MSigDB database. Module scores for each pathway were calculated with the AddModuleScore function, and results were visualized as violin plots combined with boxplots. Gene set enrichment analysis (GSEA) was performed using the clusterProfiler package, and enrichment results were presented with enrichment curves and normalized enrichment scores (NES).

### Statistical Analysis

All statistical analyses were performed using GraphPad Prism 8.0 software (GraphPad Software, San Diego, CA, USA). Data were presented as mean ± standard error of the mean (SEM). The Shapiro–Wilk test was used to assess data normality, and all datasets conformed to a normal distribution. For comparisons between two groups, an unpaired two‐tailed Student's *t*‐test was applied. For comparisons among multiple groups, one‐way or two‐way analysis of variance (ANOVA) followed by Tukey's multiple comparisons test was conducted as appropriate. A *P*‐value of less than 0.05 was considered statistically significant.

## Conflict of Interest

The authors declare no conflict of interest.

## Author Contributions

Y. L., Z. S., J.D., and Y.Z. contributed equally to this work and shared first authorship. QC and LL conceived the project and designed the experiments. YL conceived and supervised the study. YL and ZS conducted the majority of the experiments. JD and JL performed the analysis of the molecular docking simulation. YL, ZS, JD, YZ, and WC wrote the manuscript. YZ, TH, QQ, and MY participated in the animal experiments. All authors reviewed the results and approved the final version of the manuscript.

## Supporting information



Supporting Information

Supporting Information

## Data Availability

The data that support the findings of this study are available from the corresponding author upon reasonable request.
