# Supplementary material for: Puerarin Targets MIC19 to Suppress Mitochondrial Metabolism of Tumor‐Infiltrating Tregs and Enhance Anti‐tumor Immunity
Source: Adv Sci (Weinh). 2025 Nov 18;13(4):e12793. doi: 10.1002/advs.202512793 (PMC12822449; doi:10.1002/advs.202512793)
Supplement: Supplementary file 1 — Supporting Information [file ADVS-13-e12793-s002.docx]

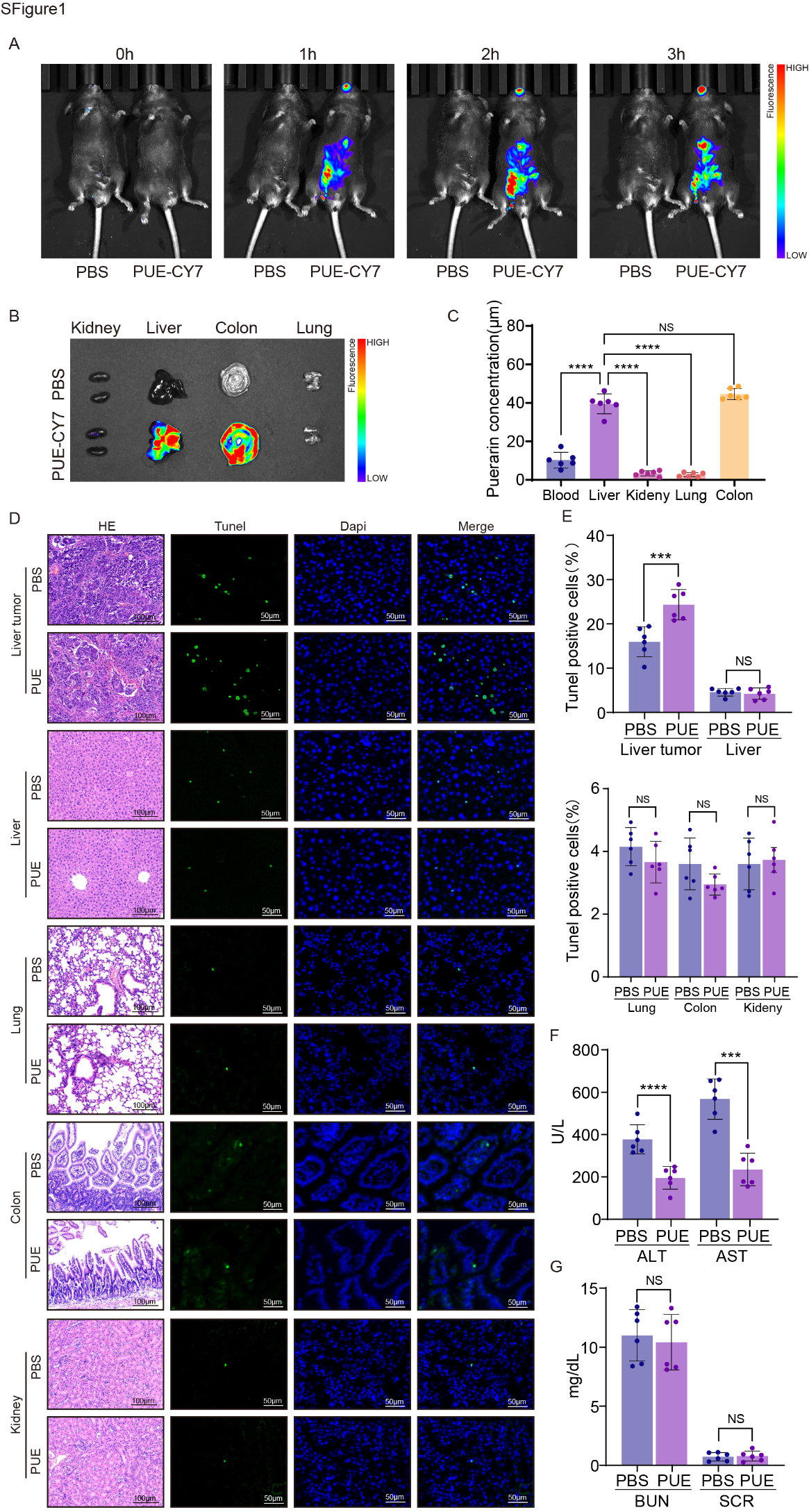


Supplementary Figure 1

(A) In vivo fluorescence imaging of mice oral gavage with PUE-CY7 or PBS at indicated time points (0–3h).

(B) Ex vivo imaging of major organs (kidney, liver, colon, lung) harvested at 3h oral gavage.

(C) Quantification of puerarin concentrations in blood, liver, kidney, lung and colon using puerarin assay kit after 4-weeks treatment of puerarin (n = 6).

(D) C57BL/6 mice were orthotopically injected with 1×10⁶ Hepa53.4 cells and treated daily with oral gavage of PUE (2.0 mg kg^-1^day^-1^) starting one-week post-injection. Representative H&E and TUNEL staining of liver tumors and major organs (liver, lung, colon, kidney) from the mice (n = 6).

(E) Quantification of TUNEL-positive cells (%) in liver tumors and normal liver, lung, colon, and kidney tissues (n = 6).

(F) Serum alanine aminotransferase (ALT) and aspartate aminotransferase (AST) levels after PUE treatment (n = 6).

(G) Blood urea nitrogen (BUN) and serum creatinine (SCR) levels were assessed to evaluate renal function after PUE treatment (n = 6).

Data are presented as mean ± standard error of the mean (SEM). *P*-values were calculated using one-way analysis of variance (ANOVA) with Tukey’s multiple comparisons in (C) and unpaired two-tailed Student’s t-test in (E–G),

**P* < 0.05; ***P* < 0.01; ****P* < 0.001; *****P* < 0.0001.


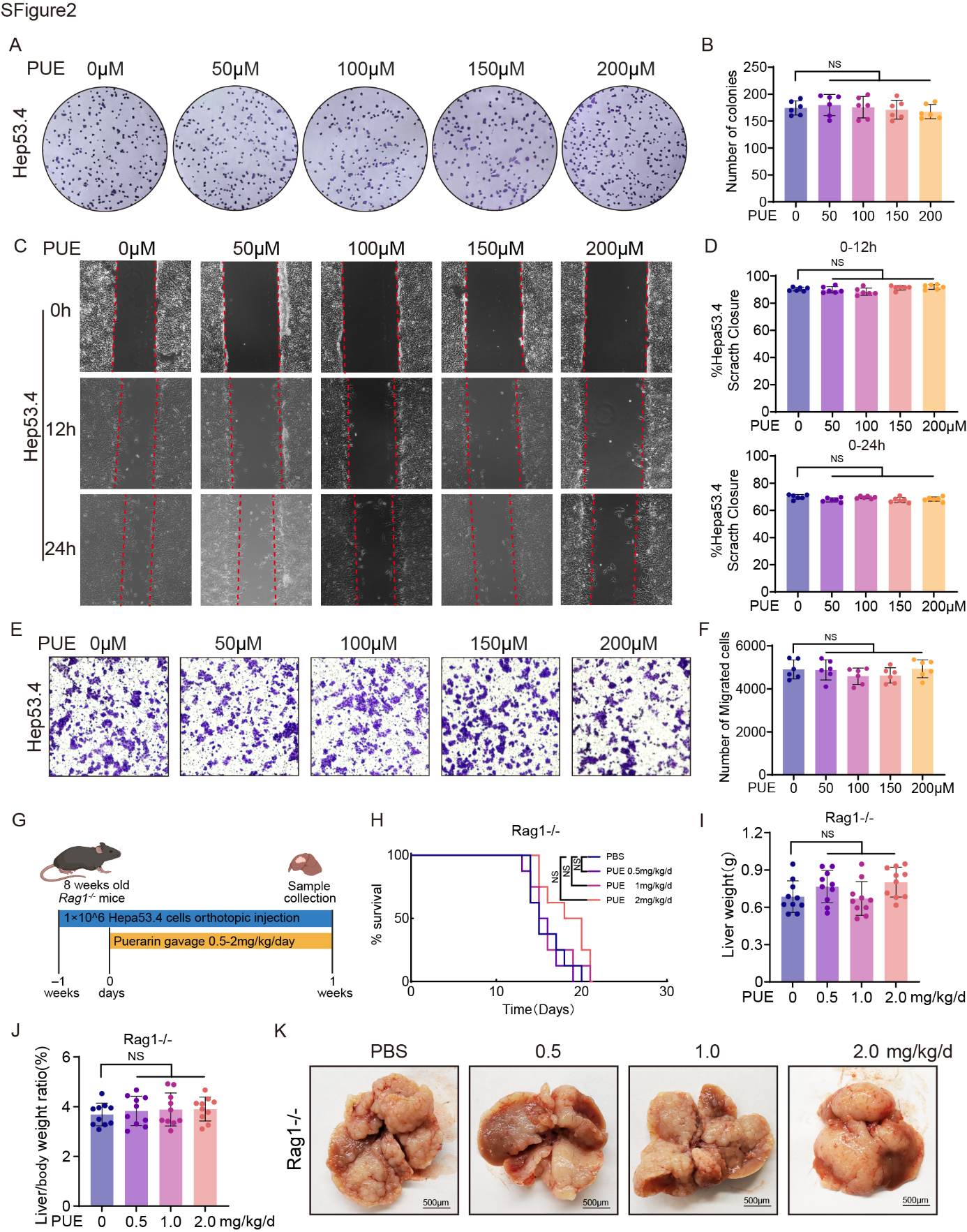


Supplementary Figure 2

(A) Representative images of colony formation assays of Hepa53.4 cells treated with increasing concentrations of puerarin (PUE) (0–200 μM) for 10 days (n = 6).

(B) Quantification of colony numbers in (A) (n = 6).

(C) Scratch wound healing assays of Hepa53.4 cells treated with PUE (0–200 μM) and photographed at 0, 12, and 24 h. Red dashed lines indicate wound edges (n = 6).

(D) Quantification of wound closure percentage at 12 h and 24 h after scratching (n = 6).

(E) Representative images of Transwell migration assays of Hepa53.4 cells treated with increasing concentrations of PUE (0–200 μM) for 24 h (n = 6).

(F) Quantification of migrated cell numbers in (E) (n = 6).

(G) Experimental scheme: Rag1^-/-^ mice orthotopically injected with 1×10⁶ Hepa53.4 cells and treated daily with oral gavage of Puerarin (PUE) (0.5–2.0 mg/kg/day) starting one-week post-injection.

(H) Kaplan–Meier survival analysis of the Rag1-/- mice treated with PBS or PUE (n = 10) (PBS vs PUE 0.5 mg kg^-1^day^-1^: hazard ratio [HR] = 1.02, 95% confidence interval [CI] = 0.38-2.71; PBS vs PUE 1.0 mg kg^-1^day^-1^: HR = 0.99, 95% CI = 0.37-2.64; PBS vs PUE 2.0 mg kg^-1^day^-1^: HR = 0.87, 95% CI = 0.33-2.32).

(I) Liver weight of the Rag1-/- mice at the experimental endpoint in each treatment group (n = 10).

(J) Liver-to-body weight ratio of the Rag1^-/-^ mice in each treatment group (n = 10).

(K) Representative image of hepatoma at 3 weeks after PUE treatment with different doses in Rag1-/- mice.

Data are presented as mean ± standard error of the mean (SEM). *P*-values were calculated using one-way analysis of variance (ANOVA) with Tukey’s multiple comparisons in (B), (D), (F), and (I–J), and the log-rank test for survival analysis in (H).

**P* < 0.05; ***P* < 0.01; ****P* < 0.001; *****P* < 0.0001.


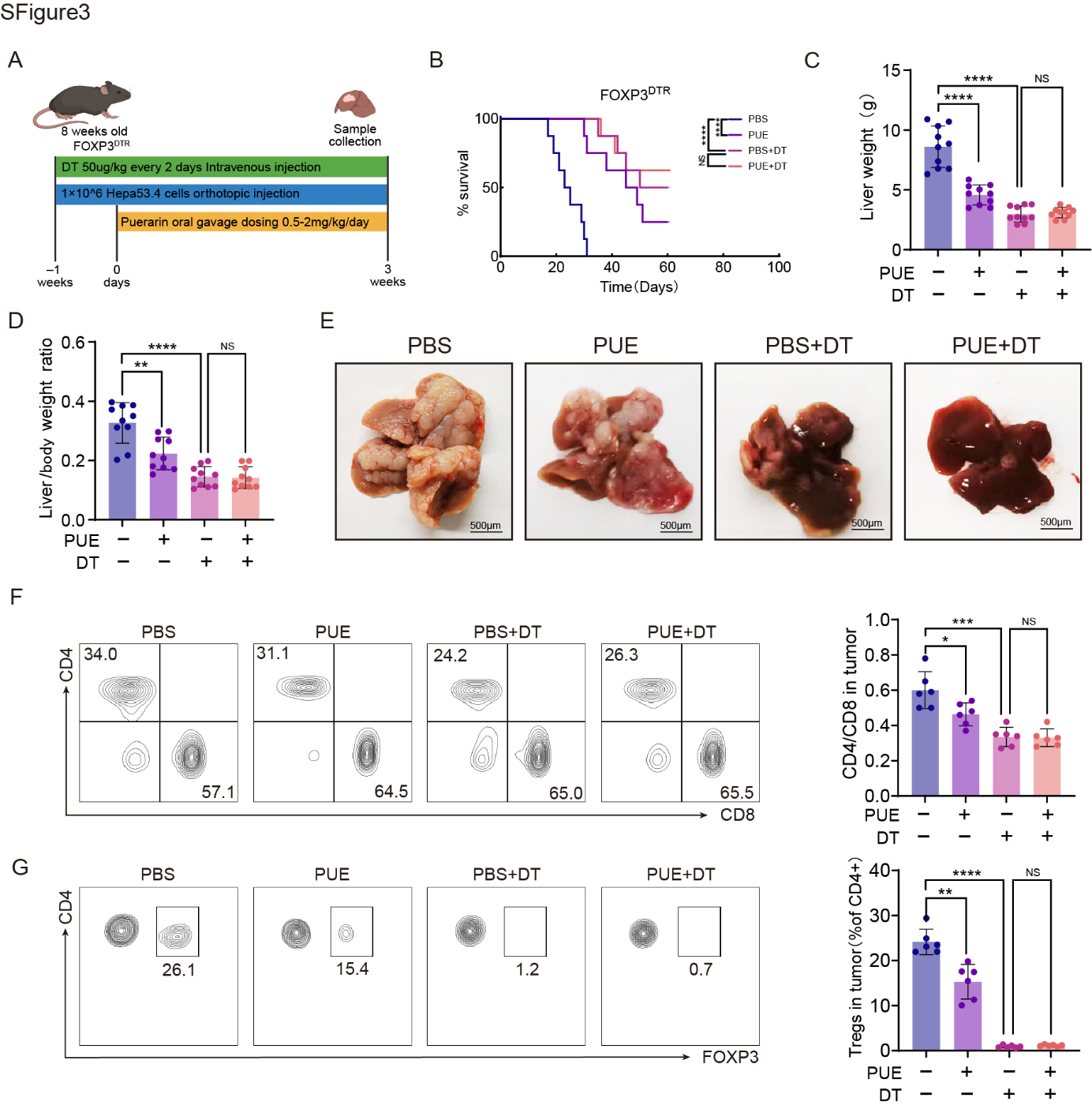


Supplementary Figure 3

(A) Experimental scheme: Foxp3^DTR^ mice orthotopically injected with 1×10⁶ Hepa53.4 cells and treated daily with oral gavage of PUE (0.5–2.0 mg/kg/day) starting one-week post-injection. Tregs were depleted, and diphtheria toxin (DT, 50 μg/kg) was intravenously injected every two days throughout the treatment period.

(B) Kaplan–Meier survival analysis of the Foxp3^DTR^ mice treated with PBS or PUE (n=10) (PBS vs PUE: hazard ratio [HR] = 0.21, 95% confidence interval [CI] = 0.083–0.55; PBS vs PBS+ DTR: HR=0.086, 95% CI = 0.027–0.28; PUE vs PUE+ DTR: HR = 0.60, 95% CI = 0.08–4.40).

(C) Liver weight of the Foxp3^DTR^ mice at an experimental endpoint in each treatment group (n = 10).

(D) Liver-to-body weight ratio of the Foxp3^DTR^ mice in each treatment group (n = 10).

(E) Representative image of hepatoma at 3 weeks after PUE treatment with different doses in Foxp3^DTR^ mice.

(F) Flow cytometry analysis of CD4⁺/CD8⁺ T cell ratios in tumor-infiltrating lymphocytes from Foxp3^DTR^ mice treated with PBS or PUE (2.0mg/kg/day) (n = 6).

(G) Flow cytometry analysis of tumor-infiltrating Tregs (Ti-Tregs) from Foxp3^DTR^ mice treated with PBS or PUE (2.0mg/kg/day) (n = 6).

Data are presented as mean ± standard error of the mean (SEM). *P*-values were calculated using a log-rank test for survival analysis in (B), and one-way analysis of variance (ANOVA) with Tukey’s multiple comparisons in (C), (D), (F), and (G).

**P* < 0.05; ***P* < 0.01; ****P* < 0.001; *****P* < 0.0001.


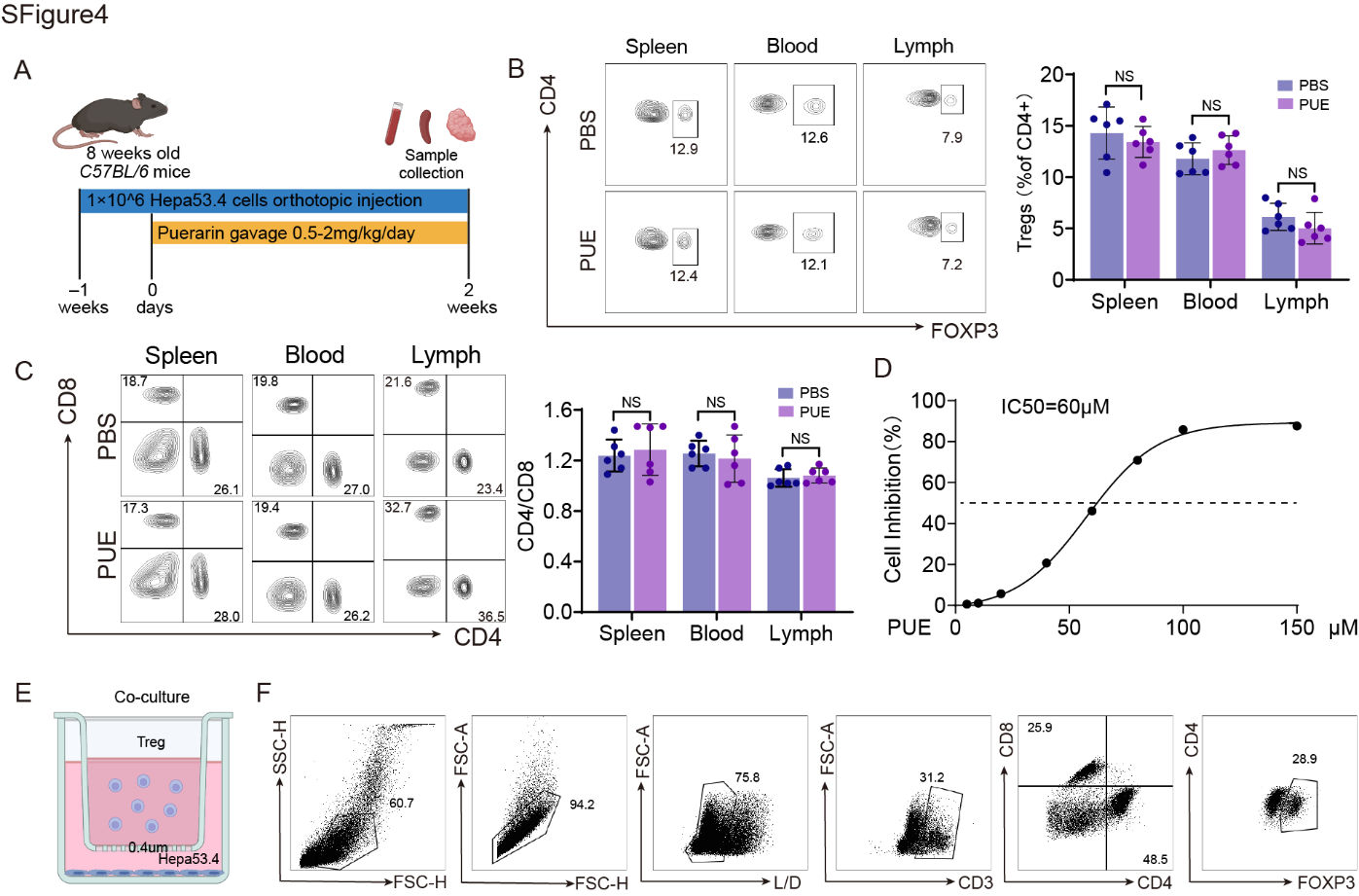


Supplementary Figure 4

(A) Experimental scheme: C57BL/6 mice were orthotopically injected with 1×10⁶ Hepa53.4 cells and treated daily with oral gavage of Puerarin (PUE) (0.5–2.0 mg kg^-1^day^-1^) starting one-week post-injection; followed by sample collection from the spleen, blood, and lymph nodes.

(B–C) Flow cytometry analysis of Tregs (B) and CD4⁺ and CD8⁺ T cells (C) in spleen, blood, and lymph nodes from mice treated with PBS or PUE (2.0 mg kg^-1^day^-1^) for 4 weeks (n = 6).

(D) Puerarin inhibits the viability of Ti-Tregs induced by co-culture with Hepa53.4 cells in a dose-dependent manner, with an IC₅₀ of approximately 60 μM.

(E) A Transwell-based indirect co-culture model to simulate tumor-infiltrating Treg (Ti-Treg) cells within the hepatocellular carcinoma (HCC) microenvironment.

(F) Gating strategy for flow cytometric identification of Ti-Tregs from murine liver cancer tissues.

Data are presented as mean ± standard error of the mean (SEM). *P*-values were calculated using unpaired two-tailed Student’s t-test in (B–C).

**P* < 0.05; ***P* < 0.01; ****P* < 0.001; *****P* < 0.0001.


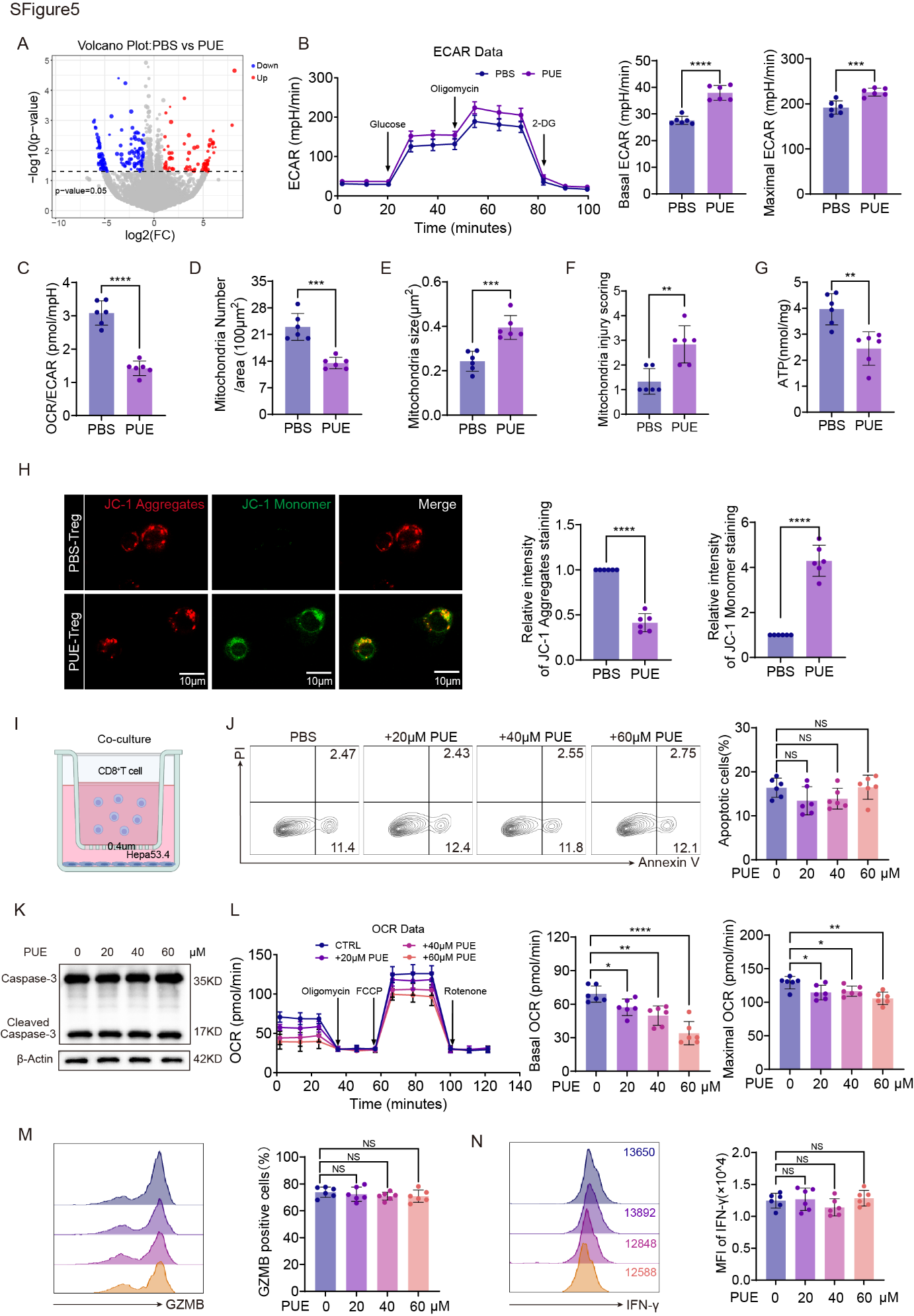


Supplementary Figure 5

(A) Volcano plot showing differentially expressed genes in mice Ti-Tregs treated with PBS or PUE in 2.0 mg kg^-1^day^-1^, highlighting significant upregulated (red) and downregulated (blue) genes (n = 3).

(B) ECAR (extracellular acidification rate) of mice Ti-Tregs treated with PBS or PUE (2.0 mg kg^-1^day^-1^) measured by Seahorse XF analyzer. Basal and maximal ECAR values were quantified (n = 6).

(C) OCR/ECAR ratio in mice Ti-Tregs treated with PBS or PUE (2.0 mg kg^-1^day^-1^) (n = 6).

(D) Quantification of mitochondrial number per 100 μm² of cytoplasmic area based on TEM images of mice Ti-Tregs treated with PBS or PUE (2.0 mg kg^-1^day^-1^) (n = 6).

(E) Measurement of average mitochondrial size in mice Ti-Tregs treated with PBS or PUE (2.0 mg kg^-1^day^-1^) from TEM sections (n = 6).

(F) Mitochondrial injury score evaluated from TEM images in mice Ti-Tregs treated with PBS or PUE (2.0 mg kg^-1^day^-1^) (n = 6).

(G) Intracellular ATP content in mice Ti-Tregs treated with PBS or PUE (2.0 mg kg^-1^day^-1^) measured by ATP assay (n = 6).

(H) Confocal images of mice Ti-Tregs stained with JC-1 dye to evaluate mitochondrial membrane potential. JC-1 aggregates (red, high potential) and monomers (green, depolarized) following treatment with PBS or PUE (2.0 mg kg^-1^day^-1^). Representative images and quantitative fluorescence intensity analysis are shown (n = 6). Scale bars=10 μm.

(I) A Transwell-based indirect co-culture model to simulate mice tumor-infiltrating CD8^+^T cells (Ti-CD8) within the hepatocellular carcinoma (HCC) microenvironment, all cells in (J–N) were co-cultured with Hepa53.4 prior to the assay to mimic the tumor immune microenvironment.

(J) Flow cytometric staining of the apoptotic mice Ti-CD8 treated with PUE (0 mM, 5 mM, 10 mM, 20 mM) for three days (n = 6).

(K) Caspase-3 and Cleaved Caspase-3 expression in mice Ti-CD8 treated with PUE (0 mM, 5 mM, 10 mM, 20 mM) for three days (n = 6).

(L) OCR of mice Ti-CD8 treated with PUE (0 mM, 5 mM, 10 mM, 20 mM) measured by Seahorse XF Analyzer. Basal and maximal OCR were quantified and compared (n=6).

(M–N) Flow cytometry analysis of GZMB and IFN-γ expression on mice Ti-CD8 treated with PUE (0 mM, 5 mM, 10 mM, 20 mM) for three days, shown as a histogram and MFI quantification (n = 6).

Data are presented as mean ± standard error of the mean (SEM). *P*-values were calculated using unpaired two-tailed Student’s t-test in (B–H) and one-way analysis of variance (ANOVA) with Tukey’s multiple comparisons in (J), and (L–N).

***P* < 0.01; ****P* < 0.001; *****P* < 0.0001.


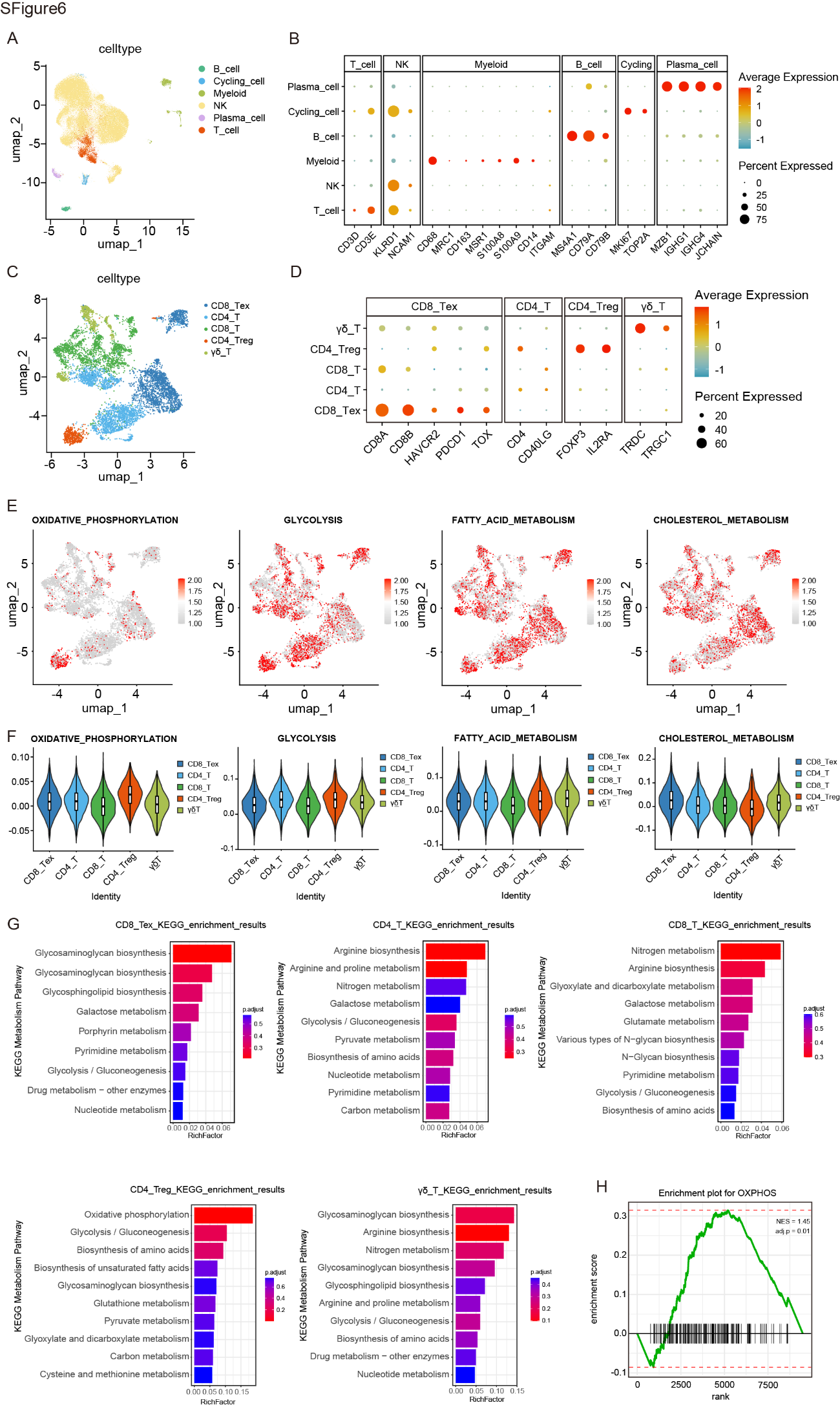


Supplementary Figure 6

(A) UMAP plot of single-cell transcriptomes from human HCC tissues (GSE162616), clustered into major immune cell types including B cells, T cells, NK cells, myeloid cells, plasma cells, and proliferative (cycling) cells.

(B) Dot plot showing the average expression and proportion of marker genes across major immune cell types.

(C) Re-clustering of T cell populations into five subtypes: CD8⁺ Tex, CD8⁺ T, CD4⁺ T, CD4⁺ Treg, and γδ T cells.

(D) Dot plot showing representative marker genes used to define T cell subtypes.

(E) UMAP visualization of enrichment scores for OXPHOS, glycolysis, fatty acid metabolism, and cholesterol metabolism at single-cell resolution.

(F) Violin plots comparing metabolic pathway scores among different T cell subsets.

(G) KEGG pathway enrichment analyses of metabolic signatures in each T cell subset.

(H) Gene set enrichment analysis (GSEA) showing that the OXPHOS gene set was significantly enriched in tumor-infiltrating Tregs compared with Tregs in blood (NES = 1.46, adjusted P = 0.01).


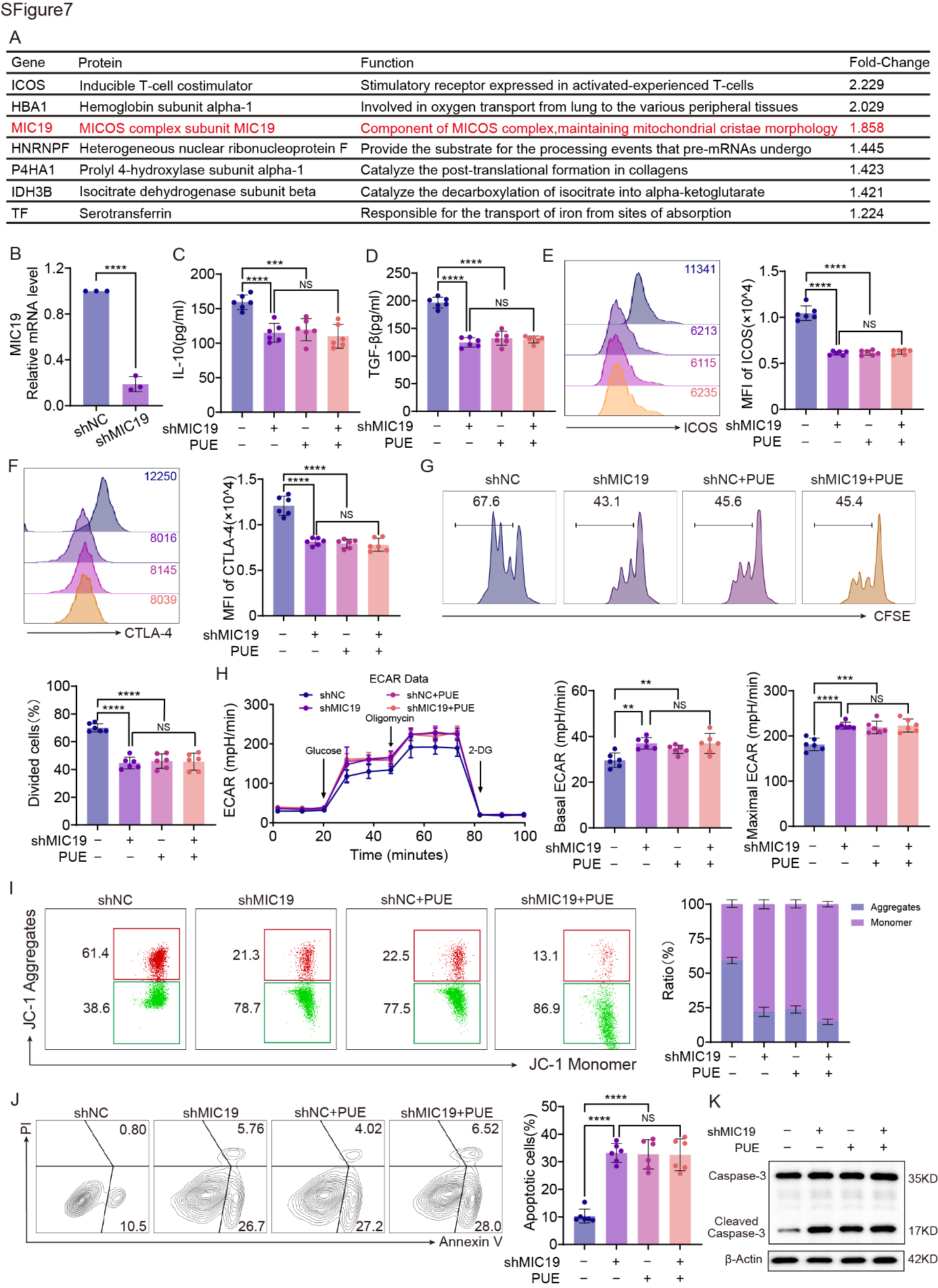


Supplementary Figure 7

(A) Table listing the 7 candidate PUE-binding proteins identified from LC-MS/MS and competition assays.

(B) RT-qPCR validation showing effective MIC19 knockdown in shMIC19-transduced mice nTregs compared to shNC control, all cells in (B-I) were co-cultured with Hepa53.4 cells prior to the assay to mimic the tumor immune microenvironment. (n=3).

(C-D) IL-10 and TGF-β levels in nTreg culture supernatants were measured by ELISA under different conditions: shNC, shMIC19, shNC+PUE, and shMIC19+PUE (n=6).

(E-F) Flow cytometry analysis of CTLA-4 and ICOS expression on mice nTregs under different conditions: shNC, shMIC19, shNC+PUE, and shMIC19+PUE, shown as a histogram and MFI quantification (n = 6).

(G) CFSE-labeled mice nTregs under different conditions: shNC, shMIC19, shNC+PUE, and shMIC19+PUE to assess proliferation, analyzed by flow cytometry (n=6).

(H) ECAR (extracellular acidification rate) of mice nTregs under different conditions: shNC, shMIC19, shNC+PUE, and shMIC19+PUE measured by Seahorse XF analyzer. Basal and maximal ECAR values were quantified (n=6).

(I) Flow cytometric analysis of mitochondrial membrane potential using JC-1 staining in mice nTregs under different conditions: shNC, shMIC19, shNC+PUE, and shMIC19+PUE. The ratio of JC-1 aggregates (red) to monomers (green) was calculated (n=6).

(J) Flow cytometric staining of the apoptotic mice shNC or shMIC19 Ti-Tregs treated with PBS or PUE (60μM) for three days (n = 6).

(K) Capase-3 and Cleaved Capase-3 expression in mice shNC or shMIC19 Ti-Tregs treated with PBS or PUE (60μM) for three days (n = 6).

Data are presented as mean ± SEM. P-values were calculated using unpaired two-tailed Student’s t-test in (B), and one-way ANOVA with Tukey’s multiple comparisons in (D-H) and (J).

**P* < 0.05; ***P* < 0.01; ****P* < 0.001; *****P* < 0.0001.


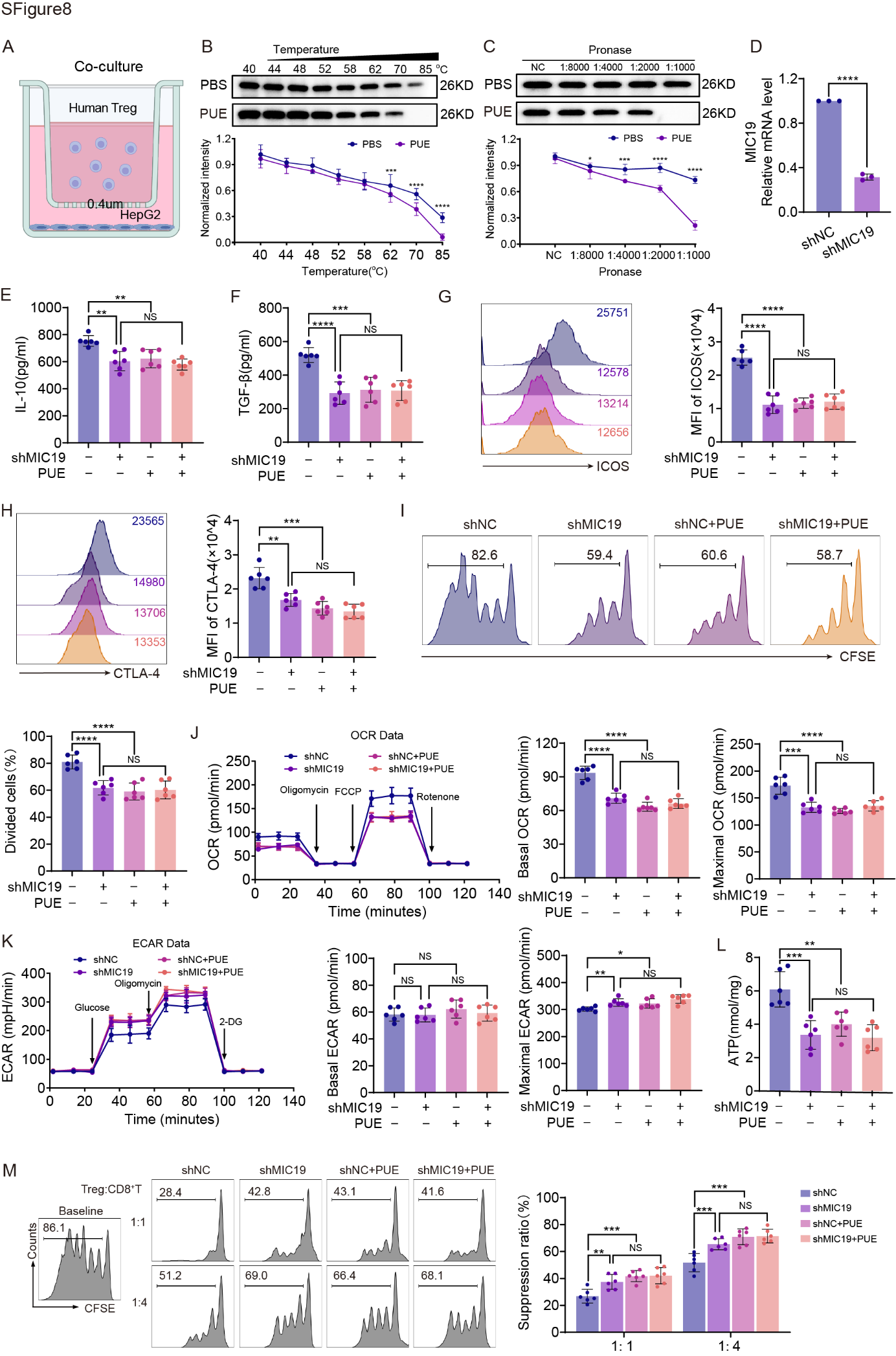


Supplementary Figure 8

(A) A Transwell-based indirect co-culture model to simulate human tumor-infiltrating Treg (Ti-Treg) cells within the hepatocellular carcinoma (HCC) microenvironment, all cells in (B-M) were co-cultured with HepG2 prior to the assay to mimic the tumor immune microenvironment.

(B) Cellular thermal shift assay (CETSA) of MIC19 protein in human Ti-Tregs treated with PBS or PUE (60μM) across a range of temperatures. Protein band intensity normalized to 40°C (n=3).

(C) Drug affinity responsive target stability (DARTS) assay of MIC19 protein in human Ti-Tregs treated with PBS or PUE (60μM), and increasing concentrations of pronase (n=3).

(D) RT-qPCR validation showing effective MIC19 knockdown in shMIC19-transduced human Ti-Tregs compared to shNC control (n=3).

(E–F) IL-10 and TGF-β levels in human Ti-Treg culture supernatants were measured by ELISA under different conditions: shNC, shMIC19, shNC+PUE, and shMIC19+PUE (n=6).

(G–H) Flow cytometry analysis of CTLA-4 and ICOS expression on human Ti-Tregs under different conditions: shNC, shMIC19, shNC+PUE, and shMIC19+PUE, shown as a histogram and MFI quantification (n = 6).

(I) Proliferation of CFSE-labeled shNC or shMIC19 human Ti-Tregs treated with PBS or PUE (60μM) for 3 days.

(J–K) OCR and ECAR of shNC or shMIC19 human Ti-Tregs treated with PBS or PUE (60μM) measured by Seahorse XF Analyzer. Basal and maximal OCR (or ECAR) were quantified and compared (n=6).

(L) Intracellular ATP levels in shNC and shMIC19 human Ti-Tregs treated with PBS or PUE (60μM) measured by ATP assay (n=6).

(M) Suppression assay: Human CD8⁺ T cells were labeled with CFSE and were co-cultured with shNC or shMIC19 human Ti-Tregs pre-treated with PBS or PUE (60μM) at indicated Treg: CD8 ratios for 3 days, proliferation was measured by flow cytometry (n=6).

Data are presented as mean ± standard error of the mean (SEM). *P*-values were calculated using unpaired two-tailed Student’s t-test in (B–D), and one-way analysis of variance (ANOVA) with Tukey’s multiple comparisons in (E–M).

**P* < 0.05; ***P* < 0.01; ****P* < 0.001; *****P* < 0.0001.


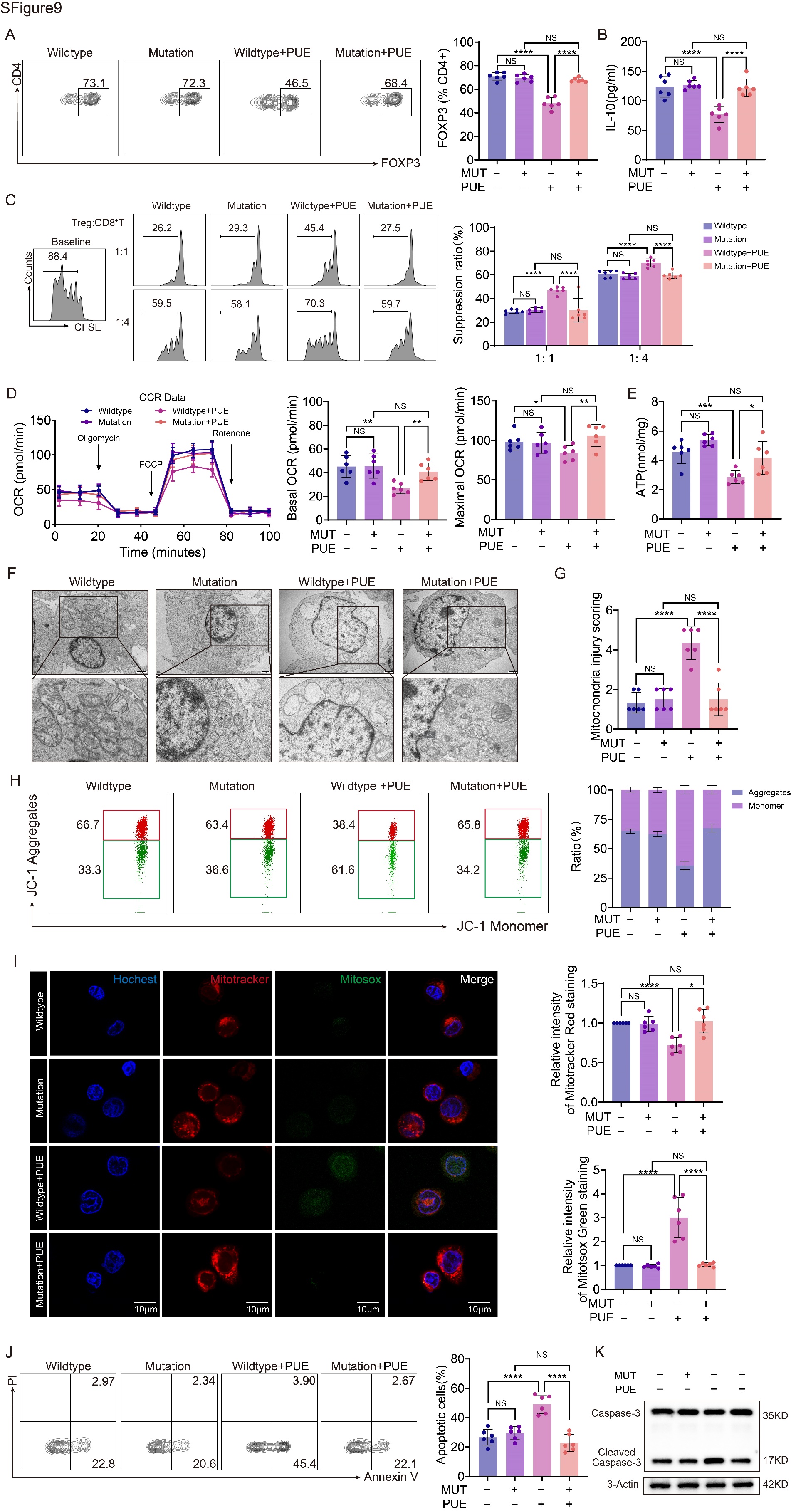


Supplementary Figure 9

(A) Flow cytometry analysis of FOXP3⁺ Treg frequency differentiated from naïve CD4⁺ T cells isolated from wild-type or MIC19^HIS180/GLN187/VAL215-Mut^ mice, stimulated with anti-CD3/CD28 magnetic beads and IL-2, and co-cultured with Hepa53.4 cells for 3 days in the presence of PBS or PUE (60 μM) (n = 6).

(B) IL-10 levels in the culture supernatants of Ti-Tregs from (A) measured by ELISA (n=6).

(C) Suppression assay of CFSE-labeled CD8⁺ T cells co-cultured with mice Ti-Tregs from (A) at indicated ratios (1:1 and 1:4). Suppression ratio was calculated based on CFSE dilution (n = 6).

(D) Seahorse analysis of the oxygen consumption rate (OCR) in tumor-infiltrating Treg (Ti-Treg) cells sorted from hepatocellular carcinoma (HCC) tissues of wild-type or MIC19^HIS180/GLN187/VAL215-Mut^ mice treated with PBS or puerarin (2.0 mg·kg⁻¹·day⁻¹). Basal and maximal OCR values are shown (n = 6).

(E) Intracellular ATP content in mice Ti-Tregs from each group measured by ATP assay (n = 6).

(F) TEM images of mitochondria in mice Ti-Tregs from each group (n = 6) (Scale bars: 1 μm).

(G) Quantification of mitochondrial injury scores based on TEM images of mice Ti-Tregs from each group (n = 6).

(H) Flow cytometric analysis of mitochondrial membrane potential using JC-1 staining in mice Ti-Tregs from each group. The ratio of JC-1 aggregates (red) to monomers (green) was calculated (n = 6).

(I) Confocal image of mice Ti-Tregs from each group stained with Hoechst, Mitotracker Red, and MitoSOX Green in each group. Quantification of fluorescence intensity of Mitotracker and MitoSOX is shown (n = 6). Scale bars = 10 μm.

(J) Flow cytometric staining of the apoptotic Ti-Tregs from wild-type or MIC19^HIS180/GLN187/VAL215-Mut^ mice treated with PBS or PUE (60μM) for three days (n = 6).

(K) Caspase-3 and Cleaved Caspase-3 expression in Ti-Tregs from wild-type or MIC19^HIS180/GLN187/VAL215-Mut^ mice treated with PBS or PUE (60μM) for three days (n = 6).

Data are presented as mean ± standard error of the mean (SEM). *P*-values were calculated using one-way ANOVA with Tukey’s multiple comparisons in (A–E), (G), (I), and (J).

**P*< 0.05; ***P* < 0.01; ****P* < 0.001; *****P* < 0.0001; NS, not significant.
