# Supplementary material for: Puerarin Targets MIC19 to Suppress Mitochondrial Metabolism of Tumor‐Infiltrating Tregs and Enhance Anti‐tumor Immunity
Source: Adv Sci (Weinh). 2025 Nov 18;13(4):e12793. doi: 10.1002/advs.202512793 (PMC12822449; doi:10.1002/advs.202512793)

**Mic19** Homo sapiens (Human)：red—151-227

MGGTTSTRRVTFEADENENITVVKGIRLSENVIDRMKESSPSGSKSQRYS

GAYGASVSDEELKRRVAEELALEQAKKESEDQKRLKQAKELDRERAAANE

QLTRAILRERICSEEERAKAKHLARQLEEKDRVLKKQDAFYKEQLARLEE

RSSEFYRVTTEQYQKAAEEVEAKFKRYESHPVCADLQAKILQCYRENTHQTLKCSALATQYMHCVNHAKQSMLEKGG

**Mic19** Homo sapiens (Human) [**Q9NX63**](https://www.uniprot.org/uniprotkb/Q9NX63/entry)

MGGTTSTRRVTFEADENENITVVKGIRLSENVIDRMKESSPSGSKSQRYSGAYGASVSDEELKRRVAEELALEQAKKESEDQKRLKQAKELDRERAAANEQLTRAILRERICSEEERAKAKHLARQLEEKDRVLKKQDAFYKEQLARLEERSSEFYRVTTEQYQKAAEEVEAKFKRYESHPVCADLQAKILQCYRENTHQTLKCSALATQYMHCVNHAKQSMLEKGG

**Mic19** [Mus musculus (Mouse)](https://www.uniprot.org/taxonomy/10090) [**Q9CRB9**](https://www.uniprot.org/uniprotkb/Q9CRB9/entry)

MGGTASTRRVTFEADENENITVVKGIRLSENVIDRMKESSPSGSKSQRYSSVYGASVSDEDLKRRVAEELALEQAKKESEHQRRLKQARDLERERAAANEQLTRAVLRERISSEEERMKAKHLARQLEEKDRVMRKQDAFYKEQLARLEERSSEFYKVTTEEYQKAAEEVEAKFKRYEYHPVCADLQTKILQCYRQNTQQTLSCSALASQYMHCVNHAKQSMLEKGG

**Mic19** Bos taurus (Bovine) [**Q5E9D3**](https://www.uniprot.org/uniprotkb/Q5E9D3/entry)

MGGTASTRRVTFEADENENITVVKGIRLSENVIDRMKETSPSGPKSQRYSGTYGASVSDEELKRRVAEELALEQAKKESENQKRLKQSKELDAEKAFANEQLTRAILRERISNEEERAKAKHLAKQLEEKDRVIKKQDAFYKEQLARLEERSSEFYKVTTEQYQKAAEEVEAKFKRYEYHPVCADLQAQILQCYRQNTQQTLSCSALASQYMRCVNQAKQSTLEKGG

MIC25/CHCHD6 [Homo sapiens (Human)](https://www.uniprot.org/taxonomy/9606) ID:[**Q9BRQ6**](https://www.uniprot.org/uniprotkb/Q9BRQ6/entry)

MGSTESSEGRRVSFGVDEEERVRVLQGVRLSENVVNRMKEPSSPPPAPTSSTFGLQDGNLRAPHKESTLPRSGSSGGQQPSGMKEGVKRYEQEHAAIQDKLFQVAKREREAATKHSKASLPTGEGSISHEEQKSVRLARELESREAELRRRDTFYKEQLERIERKNAEMYKLSSEQFHEAASKMESTIKPRRVEPVCSGLQAQILHCYRDRPHEVLLCSDLVKAYQRCVSAAHKG


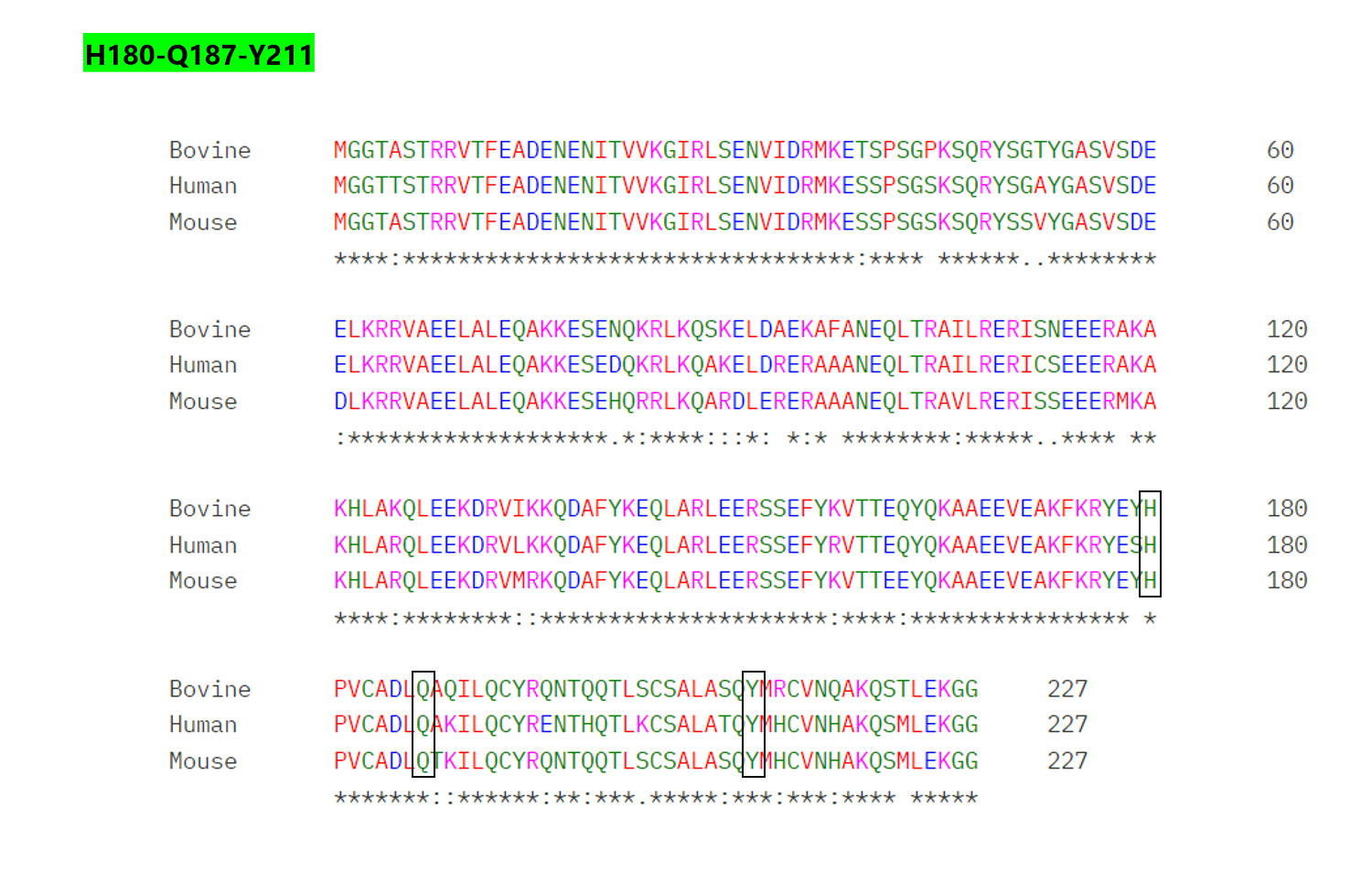

Supplement: Supplementary file 2 — Supporting Information [file ADVS-13-e12793-s001.docx]
